# Achieving Room-Temperature ppb-Level H_2_S Detection in a Au-SnO_2_ Sensor with Low Voltage Enhancement Effect

**DOI:** 10.1021/acssensors.4c00105

**Published:** 2024-05-16

**Authors:** Moumita Deb, Chia-Jung Lu, Hsiao-Wen Zan

**Affiliations:** † Department of Photonics, College of Electrical and Computer Engineering, National Yang Ming Chiao Tung University, 1001, Ta Hsueh Rd, Hsinchu 300, Taiwan; ‡ Department of Photonics, College of Electrical and Computer Engineering, National Chiao Tung University, 1001, Ta Hsueh Rd, Hsinchu 300, Taiwan; § Department of Chemistry, National Taiwan Normal University, 162, Heping East Rd., Section 1, Taipei 11677, Taiwan

**Keywords:** Au/SnO_2_ nanostructure, H_2_S, low voltage, localized dipole, ppb level, room temperature

## Abstract

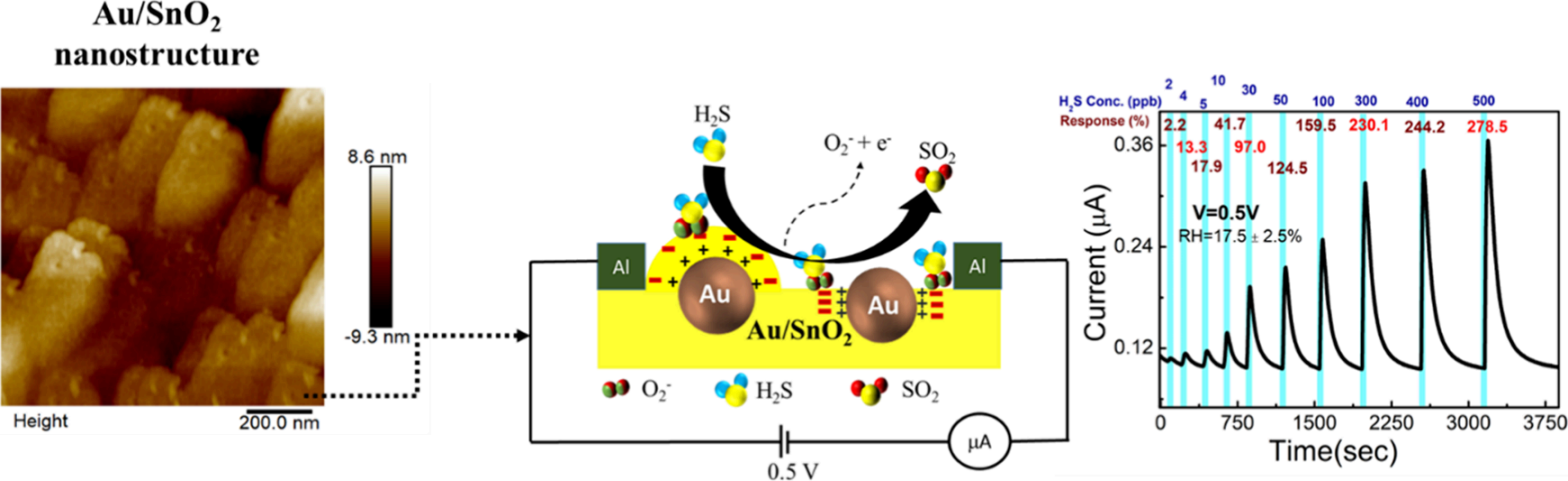

Although semiconductor metal oxide-based sensors are promising for gas sensing, low-power and room temperature operation (24 ± 1 °C) remains desirable for practical applications particularly considering the request of energy saving or net zero emission. In this study, we demonstrate a Au/SnO_2_-based ultrasensitive H_2_S gas sensor with a limit of detection (LOD) of 2 ppb, operating at very low voltages (0.05 to 0.5 V) at room temperature. The Au/SnO_2_-based sensor showed approximately 7 times higher response (the ratio of change in the current to initial current) of ∼270% and 4 times faster recovery (126 s) compared to the pure SnO_2_-based sensor when exposed to 500 ppb H_2_S gas concentration at 0.5 V operating voltage at relative humidity (RH) 17.5 ± 2.5%. The enhancement can be attributed to the catalytic characteristics of AuNPs, increasing the number of adsorbed oxygen species on sensing material surfaces. Additionally, AuNPs aid in forming flower-petal-like Au/SnO_2_ nanostructures, offering a larger surface area and more active sites for H_2_S sensing. Moreover, at low voltage (<1 V), the localized dipoles at the Au/SnO_2_ interface may further enhance the absorption of polar oxygen molecules and hence promote the reaction between H_2_S and oxygen species. This low-power, ultrasensitive H_2_S sensor outperforms high-powered alternatives, making it ideal for environmental, food safety, and healthcare applications.

Hydrogen sulfide (H_2_S) gas is toxic and flammable in ambient atmosphere.^1^ This gas is generated from mainly bacterial decomposition of organic substances, sewages, wastewater plant treatment, etc.^2^ Even at low concentrations, it can pose serious health risks. According to the American Industrial Hygiene Association (AIHA), a 100 ppb H_2_S gas exposure for one hour can pose health problems. The World Health Organization (WHO) also sets a 100 ppb time-weighted average limit for H_2_S over 24 h in their air quality guidelines.^1^ Thus, accurate ppb-level detection is essential to ensure the safety of workers in industries like oil and gas, where H_2_S may be present.^3^ On the other hand, it is an essential component in industries related to paper manufacturing,^4^ tanneries,^5^ and also food packaging.^6^ H_2_S is crucial in agriculture as a disinfectant and fertilizer.^7^ H_2_S gas also functions as a breath biomarker in conditions such as asthma,^8^ inflammation,^9^ and halitosis diseases.^10^ For instance, patients require detection of exhaled H_2_S concentrations at the ppb level.^11,12^ There are many analytical methods to develop an effective H_2_S gas sensor, such as optical,^13^ chromatographic,^14^ conductometric,^15^ colorimetric,^16^ acoustic,^17^ electrochemical,^18^ etc. The literature reveals that metal oxide semiconductors (ZnO, SnO_2_, ITO, WO_3_, CuO, NiO),^19^ conducting polymers,^20^ and nanomaterials (CNT, GO, rGO)^21,22^ are good candidates for H_2_S sensing. A metal oxide (MOx)-based H_2_S sensor is fabricated following a simple process and exhibits high sensitivity and fast response^23^ although metal oxide (MOx) sensors endure challenges such as low selectivity, high power demand (via elevated voltage), and demanding temperatures (>100 °C).^24^ Recently, several methods are developed to fabricate efficient MOx-based sensors operated at room temperature (RT) by doping noble metals (i.e., gold, palladium, and platinum),^25^ adding polymers,^26^ or applying ultraviolet (UV) light.^27^

SnO_2_ is an n-type semiconductor with a wide energy band gap of 3.5 to 4 eV and is the most promising material for gas sensing due to its unique crystal structure and high mobility of electron.^28^ In gas sensing research, the reaction between the sensor and gas primarily takes place on the surface of the sensitive material. Thus, it is crucial to increase the specific surface area of the sensitive material to enhance the possibility of gas molecules coming into contact with it, thereby increasing the sensitivity. The use of hierarchical nanostructures, which possess a large specific surface area, multiple functional adjustment dimensions, and flexible application methods, has become prevalent in gas sensing research. Recently, gold nanoparticles (AuNPs) have been widely used to increase the specific surface area, improving sensitivity, LOD (limit of detection), response/recovery time, and operating temperature. For instance, a macroporous Au/SnO_2_-based sensor demonstrates a LOD of 7 ppb and a 7-fold increase in response toward 200 ppb NO_2_ gas compared to pure SnO_2_ under UV illumination at RT.^29^ Similarly, a Au/SnO_2_ thin film-based sensor exhibits a 67.4% response, whereas a pure SnO_2_-based sensor displays only a 30.81% response toward 100 ppm of CO gas within a temperature range of 25–300 °C.^30^ Many recently reported Au/SnO_2_-based gas sensors can be found in Table S1.^29−34^ Overall, these results indicate that the flower-petal-like Au/SnO_2_ nanostructure is a promising sensing material for gas detection. Therefore, research efforts are aimed at achieving the best performance of SnO_2_-based H_2_S gas sensors through preferable modifications and structures. Table S2(33−40) presents a comparison of recent SnO_2_ composite-based H_2_S gas sensors with different key parameters, such as working temperature, gas concentration, gas response, response/recovery time, and LOD. It is observed that sensors based on different types of nanostructures, such as nanotubes,^34^ nanoporous materials,^35,39^ nanofibers,^33,37^ and nanoflakes,^38^ demonstrate superior gas sensing performance due to their large surface area. For instance, mesoporous SnO_2_^36^ and ZnO-SnO_2_ nanofiber-based^37^ H_2_S sensors exhibit low LODs of 0.5 ppb (at 92 °C) and 10 ppb (at 350 °C), respectively. Additionally, SnO_2_/rGO/PANI nanocomposite (at RT)-,^35^ Co and N-doped GQDs/SnO_2_ (at 260 °C) mesoporous microsphere-,^39^ and Cu-doped SnO_2_/rGO nanocomposite (at 120 °C)^40^-based sensors also show very low LODs of 50 ppb toward H_2_S gas. It is worth noting that the most efficient sensors operate at high operating temperatures. In this work, ppb-level H_2_S detection can be achieved at RT.

When facing the request to reduce the carbon emission, developing sensors for IoT technology should consider sensor operation at RT and also at low voltage.^41^ Thus, some recent research efforts also focus on low voltage metal oxide-based H_2_S sensors with low LOD. For example, a carbon dot-decorated MgO-based H_2_S sensor has been shown to work at a temperature of 30 °C and operating voltage of −0.7 V.^42^ Furthermore, a chitosan-CuO hybrid nanocomposite thin film-based sensor operated at 0.5 V has demonstrated a response to H_2_S gas at 40 °C.^43^ However, ppb-level H_2_S sensors operated at low voltage (i.e., < 1 V) and at RT are still not yet reported.

In this work, to achieve a low-powered sensor to detect low LOD H_2_S gas, we investigate the voltage effect as well as the relative humidity (RH) effect in a conductometric Au/SnO_2_-based sensor. The Au/SnO_2_-based sensor was on a glass substrate with 200 μm spacing interdigitated aluminum (Al) electrodes without using expensive and laborious lithographic techniques. The pristine SnO_2_ was prepared by a simple sol–gel technique without any matrix. To improve the response, LOD, and recovery time, we added Au nanoparticles prepared from HAuCl_4_. We also found the key that achieving a good response involves controlling low RH and operating at a low voltage. This can create unoccupied H_2_S reacting sites and utilize the dipoles at the Au/SnO_2_ interface to promote oxygen absorption, thus improving sensing response. In conclusion, this work presents a simple fabrication process, with the sensor operating at low temperatures (24 ± 1 °C) and low operating voltages (0.05 and 0.5 V), resulting in a low LOD for H_2_S gas detection at 17.5 ± 2.5% RH. Table S2 also suggests that our proposed sensor delivers promising performances.

## Experimental Procedure

Pristine 0.2 M SnO_2_ was prepared by using a simple sol–gel process. Then, 10 wt % AuNPs (10–20 nm)^44^ solution was added to the SnO_2_ solution and subjected to ultrasonication for 30 min. The Au/SnO_2_ solution was spin-coated and annealed at 450 °C for 2 h. Finally, the aluminum electrode was deposited on top of the film. Additional details regarding AuNPs preparation (S1, Figure S1), instruments (S2), Au/SnO_2_ thin film preparation (S3, Figure S2a), sensor fabrication (S3, Figure S2b), gas sensing measurement system^45^ (S4, Figure S3), and the calculated gas concentration information (S5, Tables S3 and Table S4) can be found in the Supporting Information.

## Results and Discussion

### Structural and Morphological Characterization

The XRD results (S6) confirm the presence of crystalline cassiterite^46^ SnO_2_ and the fcc face^47^ of Au in the Au/SnO_2_ film (Figure S4a). Additionally, the band gap energy (3.9 eV) of Au/snO_2_ calculated from absorption spectra (Figure S4b) also confirms the presence of cassiterite SnO_2_ with an energy gap of 3.6–4 eV.^48^

**Figure 1:**
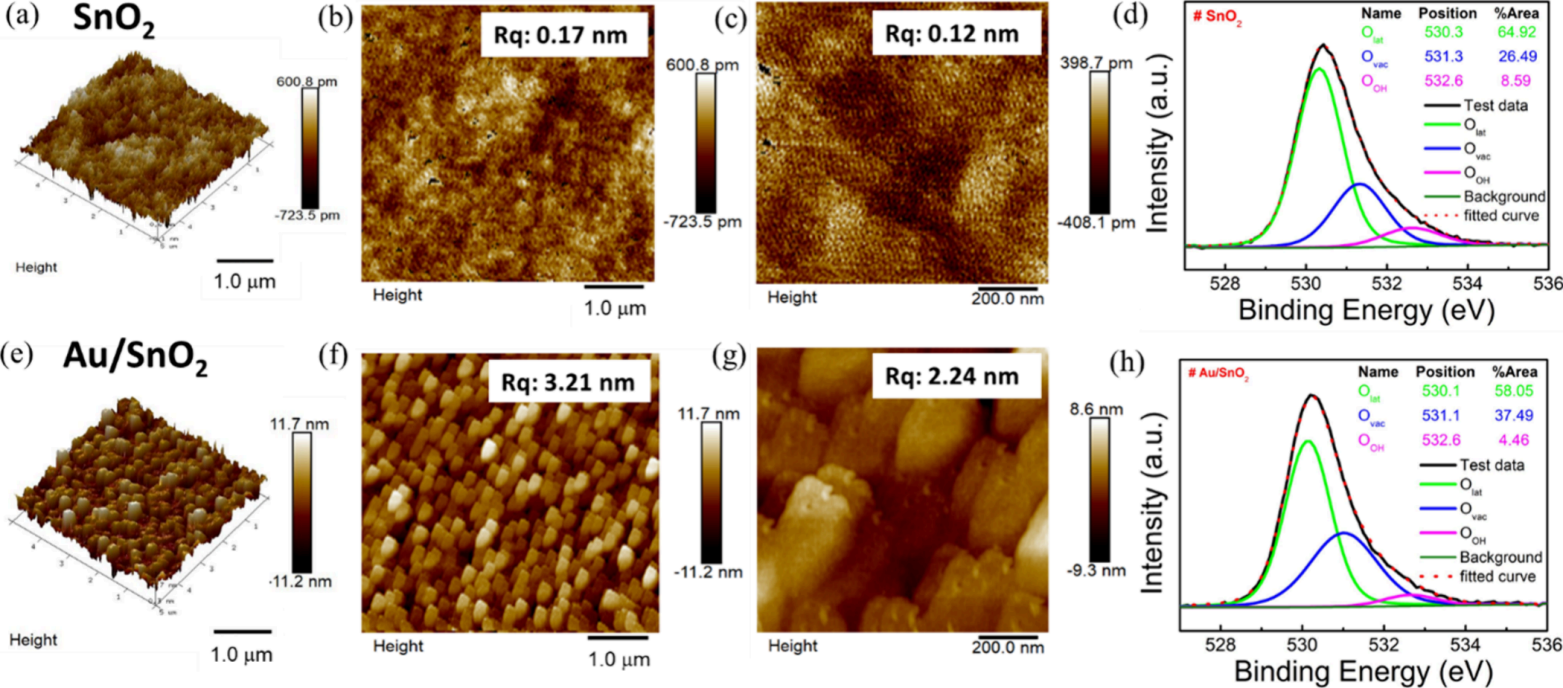
(a) 3D (5 × 5 μm^2^), (b) 2D (5 × 5 μm^2^), (c) 2D (1 × 1 μm^2^) AFM images, (d) XPS O 1s data of SnO_2_, and (e) 3D (5 × 5 μm^2^), (f) 2D (5 × 5 μm^2^), and (g) 2D (1 × 1 μm^2^) AFM images, and (h) XPS O 1s data of the Au/SnO_2_ film.

To understand the surface morphology, we conducted AFM analyses. We obtained a homogeneous Au/SnO_2_ film (Figure 1e–g) with a high surface roughness (*R*_q_ = 2.2 to 3.2 nm) and distinctive flower-petal-like nanostructures, while the pure SnO_2_ film (Figure 1a–c) has low surface roughness (*R*_q_ = 0.1 nm) in both 2D and 3D AFM images.

The XPS results prove the presence of Sn^4+^ and Au 4f peaks (S7, Figure S5).^29,32^ This substantial increase in oxygen vacancies in the Au/SnO_2_ (Figure 1h) sample compared to pure SnO_2_ (Figure 1d) indicates that the presence of AuNPs enhances the gas sensing material’s adsorption capacity for analyte gas molecules by increasing oxygen vacancy sites.^32^ Having obtained information about the proper structure and elemental composition of the SnO_2_ and Au/SnO_2_ sensing films, our focus on the gas sensing performance.

### Importance of Low Operating Voltage in Gas Sensing Performance

**Figure 2:**
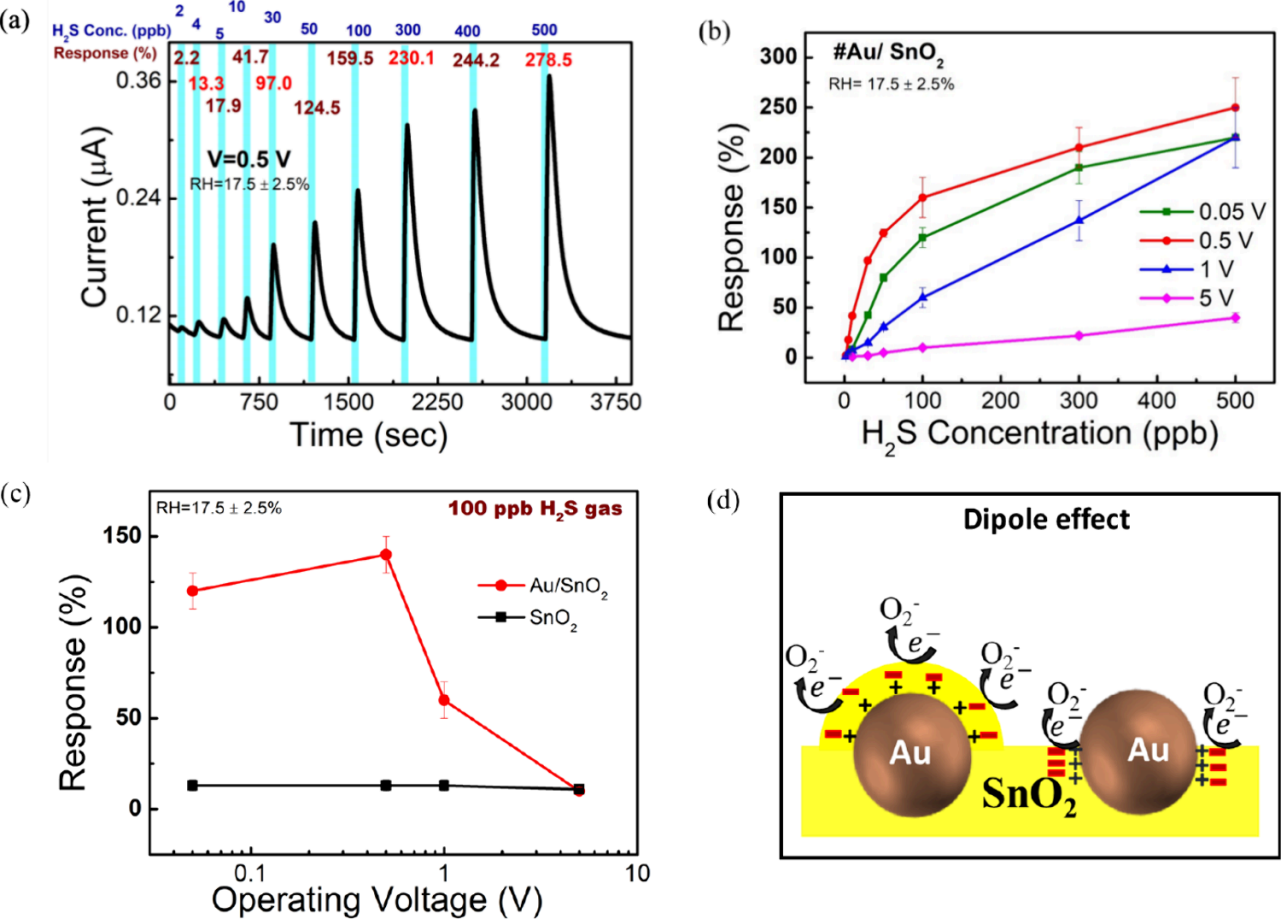
(a) Dynamic response of H_2_S gas at an operating voltage of 0.5 V. (b) Response vs H_2_S gas concentration curve of Au/SnO_2_-based sensor at different operating voltage. (c) Response vs operating voltage curve of SnO_2_ and Au/SnO_2_ based sensor at 100 ppb H_2_S gas concentration and (d) dipole effect of the Au/SnO_2_ interface at low operating voltage (<1 V).

In this section, we discuss the gas sensing performance of a Au/SnO_2_-based gas sensor operated at different working voltages. First, we analyzed the current–voltage (*I*–*V*) characteristic measurement at 17.5 ± 2.5% RH to understand the sensor’s conductivity. It was observed that the sensor achieved a microampere (μA) level current (Figure S6a) at a very low operating voltage (<1 V). This current level is suitable for our gas sensing measurement. Therefore, we decided to operate the sensor at a low operating voltage of ≤1 V and also compare with a high operating voltage of 5 V. To examine the dynamic response of the sensor under different H_2_S gas concentrations, we operated the sensor at voltages of 0.05, 0.5, 1, and 5 V (Figure 2a, and Figure S6b–d). The real-time measurement of gas response to H_2_S concentrations ranged from 2 to 500 ppb at different operating voltages. The base current levels of the sensors in ambient air were measured as 0.01 μA (at 0.05 V), 0.1 μA (at 0.5 V), 0.15 μA (at 1 V), and 5.75 μA (at 5 V). It was evident that the presence of H_2_S gas gradually increased the current level, indicating that H_2_S acts as a reducing gas in the n-type Au/SnO_2_ semiconductor-based gas sensor. Moreover, the LOD of the sensor at different operating voltages were experimentally determined as 5 ppb (at 0.05 V), 2 ppb (at 0.5 V), 2 ppb (at 1 V), and 50 ppb (at 5 V), with corresponding responses of 2.3%, 2.2%, 1.1%, and 4.6%, respectively.

The response vs gas concentration calibration curve (Figure 2b) reveals that the sensor operated at 0.05 and 0.5 V demonstrates a linear dynamic response at low H_2_S concentrations (<100 ppb) and saturates at high concentration. On the other hand, the sensor operated at 1 V exhibits a linear response ranging from 2 to 500 ppb of H_2_S gas concentration. Additionally, the sensor operated at 5 V showed a linear response from 50 to 500 ppb. Among them, the 0.5 V operated sensor exhibits the highest response (250 ± 30%) toward 500 ppb H_2_S gas, surpassing the responses of the 0.05 V (220 ± 30%), 1 V (230 ± 30%), and 5 V (33 ± 5%) operated sensors. This plot also clearly indicates that responses are significantly higher at low operating voltages (<1 V) compared to high operating voltages (5 V). The response was measured by the ratio of change in the current (*I*) to initial current (*I*_initial_).^15,49^
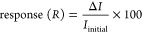
1

The response and recovery (*T*_90_) times along with the gas response at different H_2_S concentrations under various operating voltages (0.05 to 5 V) with a 30-s gas injection time are presented in Table S5. It is also noticed that higher operating voltages (0.5 and 1 V) promote faster desorption of gas molecules (faster recovery) from the sensing material surface, facilitated by increased electrical field strength. Upon cessation of H_2_S exposure, the increased electron concentration leads to the formation of O_2_^–^ to restore the sensor to its original state. Additionally, it was observed that our proposed sensor (Figure 2a) does not reach saturation after a 30 s injection. Hence, determining the exact response and recovery (*T*_90_) times alongside the gas response becomes crucial (Table S6). Consequently, the injection time (Figure S7) was extended from 30 to 300 s for 100 ppb H_2_S gas to assess the saturation period. It was observed that the sensor reached its maximum response (733%) after 180 s of injection and began to saturate. Furthermore, it was noted that at injection times of 180, 240, and 300 s, the sensor exhibited similar responses (733%) with consistent response and recovery times (110/130 s). This result depicts an accurate response and recovery time.

The observation of the sensor’s behavior at different operating voltages provides valuable insights into its response and sensitivity. Notably, the Au/SnO_2_-based sensor exhibits significantly higher response at low operating voltages (<1 V) compared to higher ones. In contrast, the SnO_2_-based sensor maintains a consistent response across varying operating voltages. This intriguing behavior may be attributed to localized dipoles at the Au nanoparticles (NPs) and SnO_2_ interface.^50−52^ A comparative analysis investigated the Au/SnO_2_ dipole effect and the influence of operating voltage on the sensor’s response to 100 ppb H_2_S gas. In the response vs operating voltage curve (Figure 2c), the SnO_2_-based sensor maintains a consistent response at 13 ± 3% across different voltages (Figure S8a), whereas the Au/SnO_2_-based sensor demonstrates varying responses at different voltages. The proposed plausible dipole effect at the Au/SnO_2_ interface was depicted in Figure 2d. The presence of dipoles at Au/SnO_2_ interfaces may enhance the adsorption of oxygen molecules on the sensor surface. Further discussion on the mechanism is provided in the relevant section.

### Comparison of Pure SnO_2_ and Au/SnO_2_-Based Sensors

**Figure 3:**
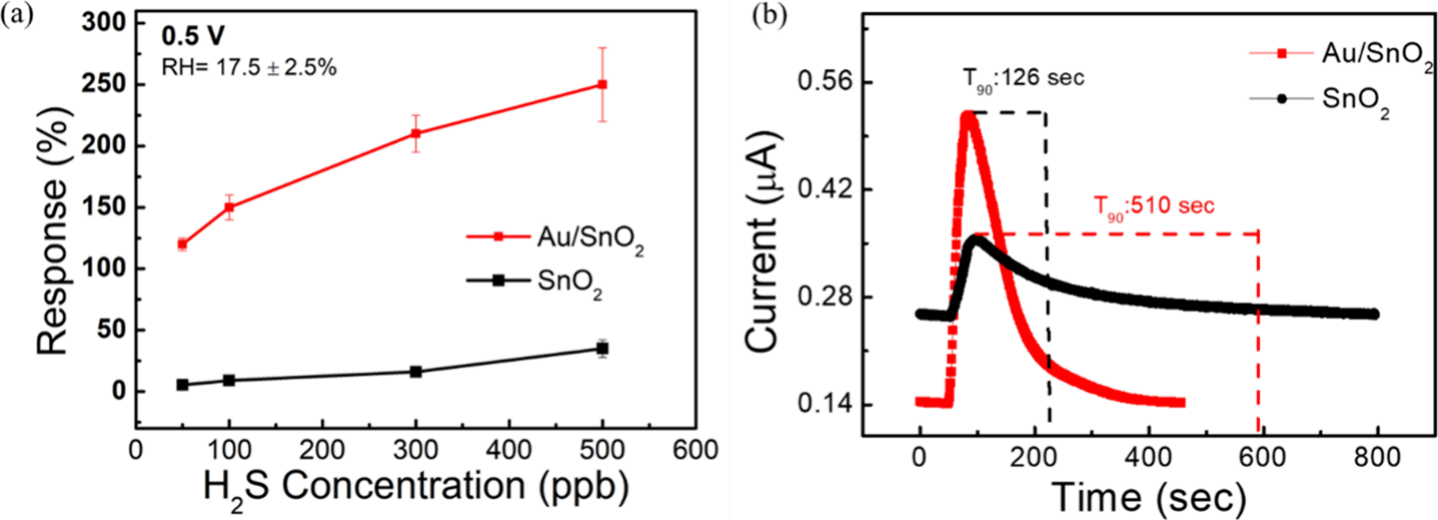
Comparison of pure SnO_2_ and 10 wt % Au/SnO_2-_based H_2_S gas sensor. (a) Response vs gas concentration calibration curve and (b) recovery time at 500 ppb H_2_ S gas concentration.

At a fixed operation voltage (0.5 V), we then investigated the AuNP effect by comparing the gas response and recovery time (*T*_90_) of the Au/SnO_2_-based sensor and the pure SnO_2_-based sensor. The presence of AuNPs significantly increases the responses toward H_2_S gas. At 500 ppb, the responses of H_2_S gas is 35 ± 7% (@ pure SnO_2_) and 250 ± 30% (@ Au/SnO_2_), respectively (Figure 3a). Additionally, AuNPs also improve the recovery time for H_2_S gas. The recovery time (T_90_) for Au/SnO_2_ and pure SnO_2_-based sensors at 500 ppb H_2_S gas are 126 and 510 s, respectively (Figure 3b). The response time was fixed at 30 s for every sensor, which is equivalent to the gas injection time. It is clear that Au/SnO_2_ improves the sensing response by more than 7 times and the recovery time by more than 4 times. It is also noticed that the calibration curves of Au/SnO_2_ is steeper than pure SnO_2_ curve. As reported in prior works, the improved performance is due to the catalytic properties in AuNPs. When AuNPs were integrated into a SnO_2_ matrix, they can enhance the sensor’s response to certain gases like H_2_S.^33,34^ AuNPs can facilitate the adsorption and dissociation of H_2_S molecules on the sensor surface, increasing the number of active sites available for gas interaction. This catalytic effect can lead to a faster and more significant response to H_2_S, resulting in a steeper calibration curve. The *I*–*V* and dynamic real-time response curve of the SnO_2_-based sensor are shown in Figure S8b,c. It is notable that (Figure S9) the 10 and 15 wt % AuNPs solutions in SnO_2_ show similar and the highest response compared to other conditions to 500 ppb H_2_S gas. Hence, we selected the 10 wt % AuNPs for further study. To determine the minimum detection limit of the H_2_S gas sensor, the LOD measurement is carried out in the next section.

### LOD, LOQ, and Sensitivity measurement

In a previous section, it was clarified that the 0.5 V operated Au/SnO_2_-based sensor is experimentally capable of detecting H_2_S gas concentration as low as 2 ppb. As a result, the interest are lies in determining the sensor’s practical LOD, which can be theoretically calculated using linear fitted function. The LOD can calculated by the standard deviation (Sy) of the response curve and the slope of the calibration curve (S) (eq 2).^53^ The Sy and S data can be obtained directly from the calibration curve origin.
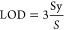
2

**Figure 4:**
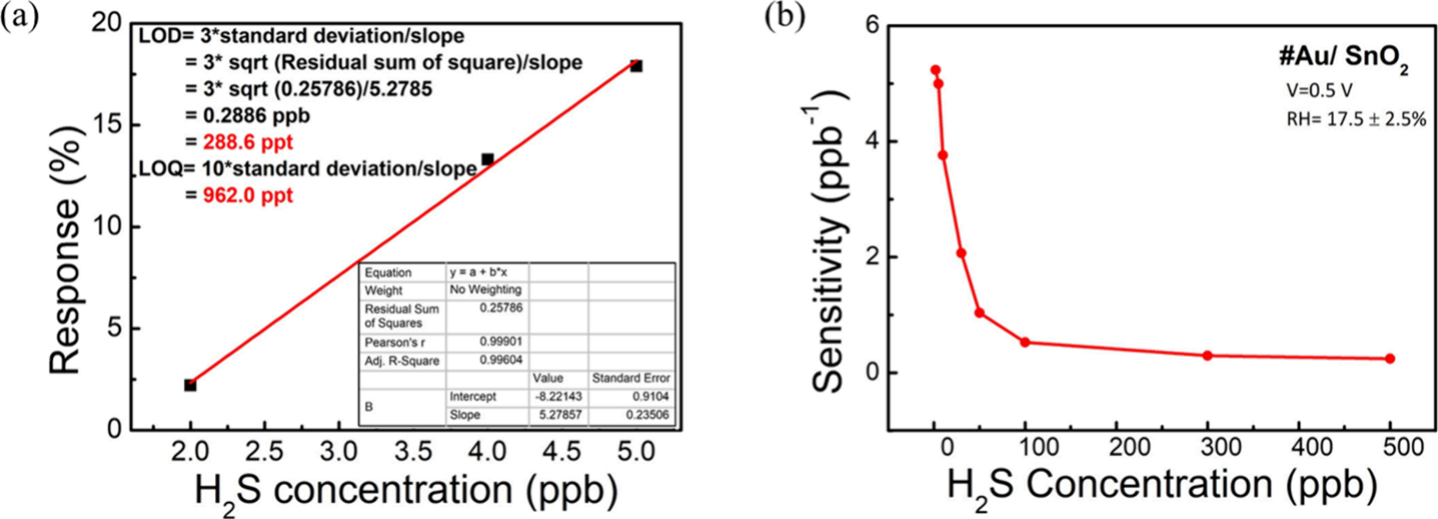
(a) LOD and LOQ and (b) sensitivity curve of the Au/SnO_2_-based H_2_S sensor at an operating voltage of 0.5 V.

To check the LOD (Figure 4a) of the sensor, three low concentrations of H_2_S gas (2, 4, and 5 ppb) were used. The fitted calibration curve demonstrates good linearity, with an *R*^2^ value of 0.99604. The calculated LOD is 288.6 ppt, which is a challenging result compared with the literature (Table S2).

LOQ (limit of quantification) is the lowest analyte concentration reliably measurable with acceptable accuracy and precision, determined akin to LOD but with higher analyte levels for quantification, and is vital for precise concentration determination, e.g., in regulatory compliance, quality control, or research. According to IUPAC, LOQ is defined as
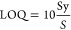
3

The calculated LOQ (Figure 4a) is 962 ppt. Therefore, we can conclude that this low-voltage operated (0.5 V) sensor exhibits the novelty of detecting H_2_S gas at the parts per trillion level, along with fast response and recovery at RT.

On the other hand, sensitivity is an important parameter of the sensor, which can determine the applicability of the sensor by indicating the sensitive zone. According to IUPAC, the sensitivity of the sensor is defined as the change in response (*R*) with respect to the H_2_S concentrations (*c*).^54^
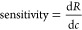
4

The sensitivity vs gas concentration plot (Figure 4b) reveals that the sensitivity of the sensor is somewhat constant from 500 to 100 ppb H_2_S, i.e., the response is linear in this range of concentration. On the other hand, the sensitivity increases sharply as the concentration of gas decreases after 100 ppb, indicating higher sensitivity of the sensor at lower concentrations, i.e., the change in response with respect to gas concentrations is higher at lower concentrations. This implies that the resolution of concentration is higher at lower concentrations. According to this sensitivity curve, the linear region (100 to 500 ppb) demonstrates excellent suitability for environmental monitoring. Conversely, the lower concentration range (<100 ppb) offers higher resolution and holds potential for detecting oral diseases like halitosis, gingivitis, and periodontitis through breath analysis.^55^ We conducted measurements (Figure S10) to observe the relative humidity effect on the sensor. Notably, the sensor exhibits a better response to H_2_S gas at low RH levels (20%). Details on humidity effects are given in Supporting Information (S8). Our low-humidity-operated H_2_S sensor at room temperature demonstrates outstanding sensing performance, rivaling traditional high-temperature processes. The reliability of the sensor will be further discussed in the next section.

### Selectivity and Repeatability Test

**Figure 5:**
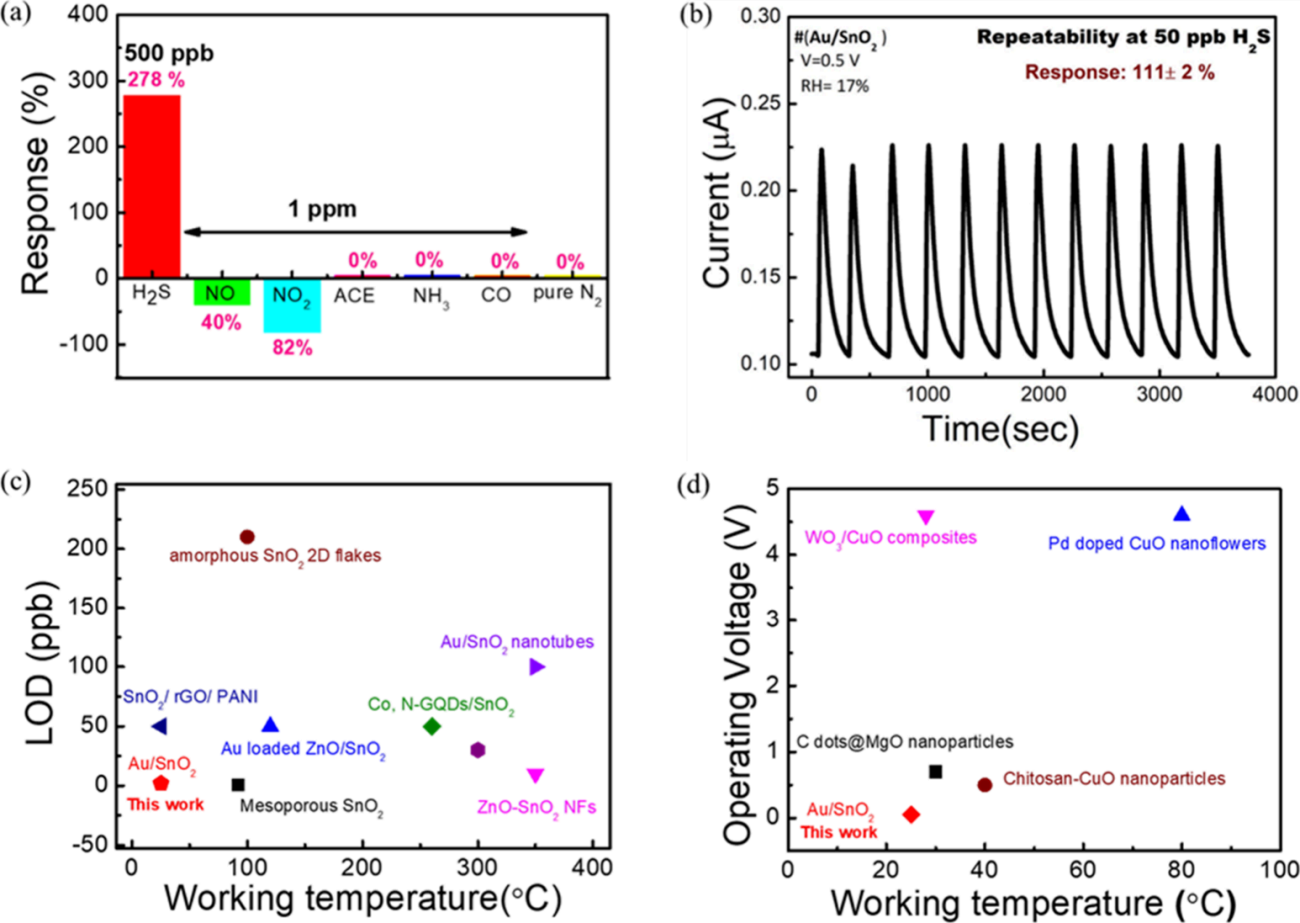
(a) Selectivity under different gas concentration and (b) 12 times repeatability curve of Au/SnO_2_ based sensor at 50 ppb H_2_S gas concentration at operating voltage of 0.5 V. (c) Comparison of the overall performance of the proposed Au/SnO_2_-based H_2_S sensor with the best low LOD H_2_S sensors (SnO_2_-based),^33−40^ and (d) low-operating voltage H_2_S sensors reported at various working temperatures (metal-oxide-based).^42,43,59,60^

To confirm the practical applicability of the sensor, we also conducted selectivity and repeatability tests. The selectivity test involved exposing the sensor to different gases (Figure 5a). We observed no response to 1 ppm acetone (ACE), ammonia (NH_3_), carbon monoxide (CO), and 25 mL of pure nitrogen (N_2_) gas. N_2_ gas was also chosen for the selectivity test because all gas cylinders contain N_2_ along with analyte gas. It is crucial to clarify that there is no effect of N_2_ (dry gas) on H_2_S gas sensing. Furthermore, the sensor exhibited responses to NO and NO_2_ gases at 1 ppm concentration, with a response of 40% and 82%, respectively, while showing a response of 278% toward 500 ppb H_2_S gas. The literature^56,57^ suggests resolving cross-sensitivity by calibrating with mixed gases. Real-time gas sensing (Figure S11) mixed 100 ppb H_2_S with NH_3_ and NO_2_. Results showed a similar response (185%) for H_2_S + NH_3_ and slightly reduced (177%) for H_2_S + NO_2_ compared to pure H_2_S. Despite NO_2_ reducing the response, it minimally impacts H_2_S sensing accuracy. The Langmuir isotherm equation^58^ may mitigate NO_2_ interference in H_2_S detection in high-NO_2_ environments. Additionally, the sensor demonstrated good repeatability (12 times) at 50 ppb H_2_S with responses of 111 ± 2% during 1 h. Thus, these results clarify that our sensor is reliable for practical applications (Figure 5b). The preliminary test for lifetime also showed that a simple thermal annealing (50 °C/15 min) can effectively prolong 30% of sensor response after 2 days. More future work is needed to develop efficient annealing conditions without degrading the response (S9, Figure S12).

### Graphical Comparison of the Proposed Sensor with the Literature

The overall performance of the proposed Au/SnO_2_-based H_2_S sensor is compared in Figure 5c,d with the best SnO_2_-based low LOD detection sensor and MOx -based low-operating voltage-operated H_2_S sensors at different working temperatures reported thus far. In Figure 5c, the *X* and *Y* axes represent the working temperature and LOD of H_2_S gas in the corresponding studies. In this figure, we demonstrate that our sensor can detect the lowest LOD (2 ppb) of H_2_S gas at the lowest operating temperature (RT: 24 ± 1 °C). Additionally, in Figure 5d, the *X* and *Y* axes represent the working temperature and operating voltage of the MOx-based H_2_S sensor in the corresponding studies. In this figure, we demonstrate that our sensor can be operated at a very low operating voltage (0.5 V) and low operating temperature (24 ± 1 °C). Our proposed sensor is marked by the red color in these two plots, indicating that our sensor not only achieves the lowest LOD but also reduces power consumption by decreasing the operating voltage and temperature. Therefore, this study clearly confirms that our proposed sensor exhibits very promising performance compared with previously reported ones.

### Sensing Mechanism

**Figure 6:**
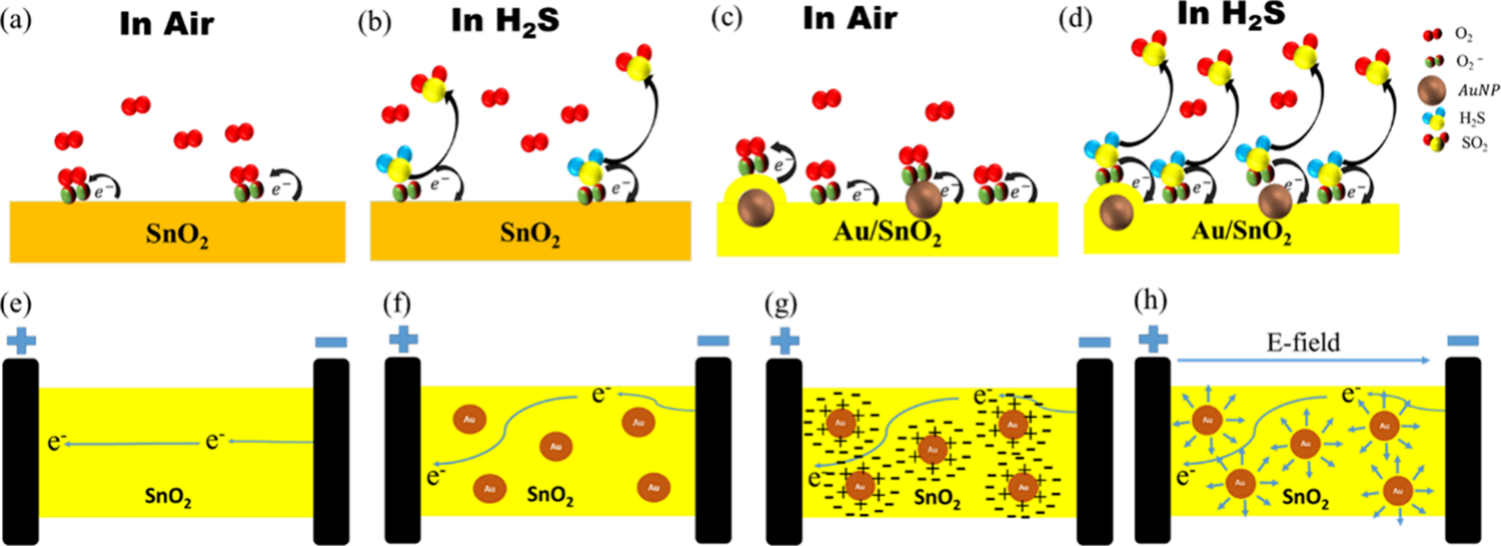
Gas sensing mechanism (a, b) on the SnO_2_ surface and (c, d) on the Au/SnO_2_ surface in air and H_2_S medium. (e) Electron flow path in the SnO_2_ Sensor. (f) Electron flow path in the Au/SnO_2_ sensor. (g) Depleted zone around Au NP. (h) Associated electric fields.

As reported in prior reports, the sensing performance of the MOx-based gas sensor generally comes from the resistance/current change caused by adsorbed oxygen on the surface of sensing materials. Additionally, oxygen vacancies play a vital role in increasing the amount of adsorbed surface oxygen. The presence of adsorbed oxygen species (O_2_^–^) improves the attachment of H_2_S gas molecules in MOx films.^36,61^ Oxygen vacancies are increased by doping of AuNPs in SnO_2,_ as is evident in the XPS data. Therefore, there is a large number of adsorbed oxygens on the Au/SnO_2_ surface. The gas sensing mechanism on the SnO_2_ and Au/SnO_2_ surfaces is depicted in Figure 6. In an air medium (Figure 6a), O_2_ molecules come in contact with the surface of SnO_2_, taking electrons from the n-type semiconductor SnO_2_ and creating O_2_^–^ at low temperatures (<100 °C).^36,61−65^ When reducing H_2_S gas interacts with the adsorbed O_2_^–^ molecules on the surface, electrons are released, increasing the electron concentration on the surface of SnO_2_ (Figure 6b). Consequently, the conductivity increases in the presence of a H_2_S gas. Furthermore, the adsorbed oxygen on the Au/SnO_2_ surface is higher compared to that on the pure SnO_2_ surface (Figure 6c), clearly indicating an increased attachment of H_2_S gas molecules on the Au/SnO_2_ surface (Figure 6d). The redox reactions of the gas sensor in air and H_2_S medium are shown below (eqs 5 and 6).^36,61−65^ The evidence of the SO_2_ product is also shown in the literature using Gas Chromatography–Mass Spectrometry measurement.^65^
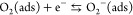
5
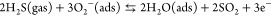
6

The enhanced response of the Au/SnO_2_ based sensor is attributed to the rise in oxygen vacancies within the film (eq 5). However, to illustrate the voltage-dependent behavior of Au/SnO_2_ at low operating voltages (<1 V), attention must be directed toward the dipole effect at the interface of Au nanoparticles and SnO_2_. To adequately explain this effect at low voltages, schematics depicting current flow between the electrodes in both the SnO_2_ and Au/SnO_2_ sensors are provided (Figure 6e–h). Figure 6e shows the path of electron flow (reverse of current) in the SnO_2_ film from the cathode to the anode. Similarly, the electron flow in Au/SnO_2_ is depicted in Figure 6f. From the comparison of these two figures, it is obvious that the voltage dependency of the response originates from the presence of the Au nanoparticles in the SnO_2_ film. The charge transfer at the Au/SnO_2_ interface will form a depletion zone in the SnO_2_ semiconductor as depicted in Figure 6g. This type of charge transfer occurs due to the high oxygen vacancy^50^ on the film formed by AuNPs. The depleted area around the Au nanoparticles are portrayed in Figure 6h. This area will interact with the ionized oxygen species and the slightly polar H_2_S molecules, increasing the response of the sensor. However, the increase in electric field between the electrodes (higher voltage: > 1 V) would suppress the electric fields in the depletion zone, resulting in the voltage-independent response. Obviously, the direction of the electric field in the depletion zone in the SnO_2_ film will be reversed in the case of no or low oxygen vacancies. However, the voltage dependency will remain the same irrespective of the direction of the electric fields in the depletion zone. At low operating voltages (0.05 and 0.5 V), localized dipoles emerge at the AuNPs/SnO_2_ interface due to the interaction between AuNPs and SnO_2_. This interaction induces charge separation (Figure 6g), resulting in the formation of localized electric dipoles (Figure 6h). These dipoles may play a dominant role in enhancing the sensor’s response, particularly for gases like H_2_S. They facilitate the adsorption and dissociation of gas molecules on the sensor surface, leading to a more pronounced response. However, when the operating voltage is increased to 1 V, the applied voltage starts to disrupt the localized dipoles, leading to a decrease in response. Further increments in applied voltage (5 V) entirely destroy the localized dipoles, causing the response due to localized polarization to vanish. As a result, the Au/SnO_2_-based sensor shows results similar to those of the SnO_2_-based sensor. However, the above-mentioned mechanisms are still plausible mechanisms. More investigation should be conducted in future work to verify the existence of interfacial localized dipoles.

In this research, we observed the improvement of sensing performances of Au/SnO_2_ film toward H_2_S gas at room temperature due to the following factors:

i. The fabricated Au/SnO_2_ thin film exhibited a nanostructure, as evidenced by the AFM images. This nanostructure offers a larger surface area for interaction between H_2_S gas molecules, leading to an improvement in gas sensing performance, including response, sensitivity, LOD, and recovery time.
ii. XPS spectra revealed the presence of oxygen vacancy defects in the film. These vacancies, along with surface adsorbed oxygen ions, influence the gas sensing properties and mechanism. This abundance of oxygen vacancies promotes a stronger electrostatic interaction between the reactive gas molecules and the Au/SnO_2_ surface, improving H_2_S sensing at RT. Additionally, the adsorbed oxygen on the oxygen-deficient surface enhances the interaction with H_2_S. Thus, the high response of the prepared Au/SnO_2_ thin films to H_2_S gas can be attributed to a significant presence of surface adsorbed oxygen species, estimated to be approximately 37.49% of the total oxygen content, based on the O 1s XPS spectrum.
iii. The choice of operating voltage in Au/SnO_2_-based H_2_S gas sensors is critical for optimizing the sensor’s response to H_2_S gas. At low operating voltage (<1 V), the presence of the dipoles at the Au/SnO_2_ interface may affect the charge distribution and the interaction with H_2_S. As a result, low operating voltage gives rise to enhanced response as well as good repeatability.

## Conclusions

An ultrasensitive H_2_S gas sensor based on Au/SnO_2_ flower-petal-like nanostructures has been successfully demonstrated. The sensor achieved revolutionary low limits of detection (LOD) of 2 ppb (experimentally) and 288.6 ppt (calculated), along with a high response of ∼270% at 500 ppb. The presence of Au nanoparticles (AuNPs) in the SnO_2_ film not only increased the gas sensing performance by 7 times but also improved the recovery time by 5 times at 500 ppb H_2_S gas compared to pure SnO_2_. The study indicates that the enhanced sensing performance is attributed to the larger surface area and the increased presence of adsorbed oxygen species on the Au/SnO_2_ film surface. Furthermore, the dipole effect at the Au/SnO_2_ interface may enhance the sensor’s performance, allowing for a low optimal operating voltage of 0.5 V at RT (24 ± 1 °C). This study also indicates that a room-temperature, low-RH-operated H_2_S sensor outperforms conventional high-temperature sensors under reduced RH conditions. In conclusion, the Au/SnO_2_ sensors are capable to serve as low power ultrasensitive H_2_S sensors and are promising for environmental, clinical, and food quality monitoring applications in the IoT era.

## Data Availability

Data will be made available on request.

## Supporting Information Available

Chemicals, characterization, gas sensing system, XRD patterns, XPS spectra, UV–vis absorption spectra, additional gas sensing data, comparison tables, gas concentration calculation and response/recovery tables for gas sensing performance is attached (PDF)

## References

[ref1] Malone Rubright S. L.; Pearce L. L.; Peterson J. Environmental toxicology of hydrogen sulfide. Nitric Oxide 2017, 71, 1–13. 10.1016/j.niox.2017.09.011. 29017846 PMC5777517

[ref2] Habeeb O. A.; Kanthasamy R.; Ali G. A.; Sethupathi S.; Yunus R. B. M. Hydrogen sulfide emission sources, regulations, and removal techniques: a review. Rev. Chem. Eng. 2018, 34, 837–854. 10.1515/revce-2017-0004.

[ref3] Marriott R. A.; Pirzadeh P.; Marrugo-Hernandez J. J.; Raval S. Hydrogen sulfide formation in oil and gas. Can. J. Chem. 2016, 94, 406–413. 10.1139/cjc-2015-0425.

[ref4] Janssen A. J.; Lens P. N.; Stams A. J.; Plugge C. M.; Sorokin D. Y.; Muyzer G.; Dijkman H.; Van Zessen E.; Luimes P.; Buisman C. J. Application of bacteria involved in the biological sulfur cycle for paper mill effluent purification. Sci. Total Environ. 2009, 407, 1333–1343. 10.1016/j.scitotenv.2008.09.054. 19027933

[ref5] Shivasankaran N.; Balan A. V.; Sankar S. P.; Magibalan S.; Dinesh C. M. Removal of hydrogen sulphide and odour from tannery & textile effluents. Mater. Today Proc. 2020, 21, 777–781. 10.1016/j.matpr.2019.07.242.

[ref6] Al Shboul A. M.; Izquierdo R. Printed chemiresistive In_2_O_3_ nanoparticle-based sensors with ppb detection of H_2_S gas for food packaging. ACS Appl. Nano Mater. 2021, 4, 9508–9517. 10.1021/acsanm.1c01970.

[ref7] Bloem E.; Haneklaus S.; Kesselmeier J.; Schnug E. Sulfur fertilization and fungal infections affect the exchange of H_2_S and COS from agricultural crops. J. Agric. Food Chem. 2012, 60, 7588–7596. 10.1021/jf301912h. 22812725

[ref8] Chung K. F. Hydrogen sulfide as a potential biomarker of asthma. Expert Rev. Respir. Med. 2014, 8, 5–13. 10.1586/17476348.2014.856267. 24308655

[ref9] Chen Y. H.; Yao W. Z.; Gao J. Z.; Geng B.; Wang P. P.; Tang C. S. Serum hydrogen sulfide as a novel marker predicting bacterial involvement in patients with community-acquired lower respiratory tract infections. Respirology 2009, 14, 746–752. 10.1111/j.1440-1843.2009.01550.x. 19659653

[ref10] Shin H.; Kim D. H.; Jung W.; Jang J. S.; Kim Y. H.; Lee Y.; Chang K.; Lee J.; Park J.; Namkoong K.; Kim I. D. Surface activity-tuned metal oxide chemiresistor: toward direct and quantitative halitosis diagnosis. ACS Nano 2021, 15, 14207–14217. 10.1021/acsnano.1c01350. 34170113

[ref11] Shahzad A. A.; Mushtaq S.; Waris A.; Gilani S. O.; Alnuwaiser M. A.; Jameel M.; Khan N. B. A Low-Cost Device for Measurement of Exhaled Breath for the Detection of Obstructive Lung Disease. Biosensors 2022, 12, 409 10.3390/bios12060409. 35735555 PMC9221323

[ref12] Liu N.; Tseng Y.; Zhang H.; Chen J.; Huang M.-H. The role of exhaled hydrogen sulfide in the diagnosis of colorectal adenoma. Can. J. Infect. Dis. Med. Microbiol. 2021, 2021, 8046368 10.1155/2021/8046368. 34900068 PMC8654565

[ref13] Zhang Y.; Wang Y.; Liu Y.; Dong X.; Xia H.; Zhang Z.; Li J. Optical H_2_S and SO_2_ sensor based on chemical conversion and partition differential optical absorption spectroscopy. Spectrochim. Acta A Mol. Biomol. Spectrosc. 2019, 210, 120–125. 10.1016/j.saa.2018.11.035. 30453187

[ref14] Feng D.; Du L.; Xing X.; Wang C.; Chen J.; Zhu Z.; Tian Y.; Yang D. Highly sensitive and selective NiO/WO_3_ composite nanoparticles in detecting H_2_S biomarker of halitosis. ACS sens. 2021, 6, 733–741. 10.1021/acssensors.0c01280. 33528988

[ref15] Nagmani; Pravarthana D.; Tyagi A.; Jagadale T. C.; Prellier W.; Aswal D. K. Highly sensitive and selective H_2_S gas sensor based on TiO_2_ thin films. Appl. Sur. Sci. 2021, 549, 149281 10.1016/j.apsusc.2021.149281.

[ref16] Wang Z.; Liu J.; Zhang L.; Nie W.; Liu J.; Yang J.; Li Y. Copper (II)-azo complex modified hydrogel: A sensitive colorimetric sensor for visual detection of H_2_S gas. Sens. Actuators B: Chem. 2023, 376, 132968 10.1016/j.snb.2022.132968.

[ref17] Li D.; Zu X.; Ao D.; Tang Q.; Fu Y.; Guo Y.; Bilawal K.; Faheem M. B.; Li L.; Li S.; Tang Y. High humidity enhanced surface acoustic wave (SAW) H_2_S sensors based on sol–gel CuO films. Sens. Actuators B: Chem. 2019, 294, 55–61. 10.1016/j.snb.2019.04.010.

[ref18] Li D.; Zu X.; Ao D.; Tang Q.; Fu Y.; Guo Y.; Bilawal K.; Faheem M. B.; Li L.; Li S.; Tang Y. High humidity enhanced surface acoustic wave (SAW) H2S sensors based on sol–gel CuO films. Sens. Actuators, B 2019, 294, 55–61. 10.1016/j.snb.2019.04.010.

[ref19] Ghaderahmadi S.; Kamkar M.; Tasnim N.; Arjmand M.; Hoorfar M. A review of low-temperature H_2_S gas sensors: Fabrication and mechanism. New J. Chem. 2021, 45, 17727–17752. 10.1039/D1NJ02468J.

[ref20] Duc C.; Boukhenane M. L.; Wojkiewicz J. L.; Redon N. Hydrogen sulfide detection by sensors based on conductive polymers: A review. Front. Mater. 2020, 7, 215 10.3389/fmats.2020.00215.

[ref21] Geng Y.; Ren Y.; Wang X.; Li J.; Portilla L.; Fang Y.; Zhao J. Highly sensitive and selective H_2_S sensors with ultra-low power consumption based on flexible printed carbon-nanotube-thin-film-transistors. Sens. Actuators, B 2020, 360, 131633 10.1016/j.snb.2022.131633.

[ref22] Chu J.; Wang X.; Wang D.; Yang A.; Lv P.; Wu Y.; Rong M.; Gao L. Highly selective detection of sulfur hexafluoride decomposition components H_2_S and SOF_2_ employing sensors based on tin oxide modified reduced graphene oxide. Carbon 2018, 135, 95–103. 10.1016/j.carbon.2018.04.037.

[ref23] Guo Z.; Chen G.; Zeng G.; Liu L.; Zhang C. Metal oxides and metal salt nanostructures for hydrogen sulfide sensing: mechanism and sensing performance. RSC Adv. 2015, 5, 54793–54805. 10.1039/C5RA10394K.

[ref24] Mirzaei A.; Kim S. S.; Kim H. W. Resistance-based H_2_S gas sensors using metal oxide nanostructures: A review of recent advances. J. Hazard. Mater. 2018, 357, 314–331. 10.1016/j.jhazmat.2018.06.015. 29902726

[ref25] Kim J.-Y.; Lee J.-H.; Kim J.-H.; Mirzaei A.; Woo Kim H.; Kim S. S. Realization of H_2_S sensing by Pd-functionalized networked CuO nanowires in self-heating mode. Sens. Actuators, B 2019, 299, 126965 10.1016/j.snb.2019.126965.

[ref26] Kumawat M.; Thapliyal D.; Verros G. D.; Arya R. K.; Barman S.; Halder G.; Shandilya P. PANI-based hydrogen sulfide gas sensors. Coatings 2022, 12, 186 10.3390/coatings12020186.

[ref27] Li Z.; Yan S.; Zhang S.; Wang J.; Shen W.; Wang Z.; Fu Y. Q. Ultra-sensitive UV and H_2_S dual functional sensors based on porous In_2_O_3_ nanoparticles operated at room temperature. J. Alloys Compd. 2019, 770, 721–731. 10.1016/j.jallcom.2018.08.188.

[ref28] Kang X.; Deng N.; Yan Z.; Pan Y.; Sun W.; Zhang Y. Resistive-type VOCs and pollution gases sensor based on SnO_2_: A review. Mater. Sci. Semicond. Process. 2022, 138, 106246 10.1016/j.mssp.2021.106246.

[ref29] Hu K.; Li Y.; Ge C.; Bai L.; Liu G.; Qiao G.; Kang S. G.; Kim E. J.; Wang M. Room-temperature ppb-level NO_2_ sensitivity of three-dimensional ordered macroporous Au-loaded SnO_2_ under intermittent UV light irradiation. Sens. Actuators B: Chem. 2023, 387, 133786 10.1016/j.snb.2023.133786.

[ref30] Rehman B.; Bhalla N. K.; Vihari S.; Jain S. K.; Vashishtha P.; Gupta G. SnO_2_/Au multilayer heterostructure for efficient CO sensing. Mater. Chem. Phys. 2020, 244, 122741 10.1016/j.matchemphys.2020.122741.

[ref31] Liu Y.; Li X.; Wang Y.; Li X.; Cheng P.; Zhao Y.; Dang F.; Zhang Y. Hydrothermal synthesis of Au@ SnO_2_ hierarchical hollow microspheres for ethanol detection. Sens. Actuators B: Chem. 2020, 319, 128299 10.1016/j.snb.2020.128299.

[ref32] Guo L.; Shen Z.; Ma C.; Ma C.; Wang J.; Yuan T. Gas sensor based on MOFs-derived Au-loaded SnO_2_ nanosheets for enhanced acetone detection. J. Alloys Compd. 2022, 906, 164375 10.1016/j.jallcom.2022.164375.

[ref33] Hung C. M.; Phuong H. V.; Van Thinh V.; Hong L. T.; Thang N. T.; Hanh N. H.; Dich N. Q.; Van Duy N.; Van Hieu N.; Hoa N. D. Au doped ZnO/SnO_2_ composite nanofibers for enhanced H_2_S gas sensing performance. Sens. Actuators, A 2021, 317, 112454 10.1016/j.sna.2020.112454.

[ref34] Jang J. S.; Kim S. J.; Choi S. J.; Kim N. H.; Hakim M.; Rothschild A.; Kim I. D. Thin-walled SnO_2_ nanotubes functionalized with Pt and Au catalysts via the protein templating route and their selective detection of acetone and hydrogen sulfide molecules. Nanoscale 2015, 7, 16417–16426. 10.1039/C5NR04487A. 26395290

[ref35] Zhang D.; Wu Z.; Zong X. Flexible and highly sensitive H_2_S gas sensor based on in-situ polymerized SnO_2_/rGO/PANI ternary nanocomposite with application in halitosis diagnosis. Sens. Actuators B: Chem. 2019, 289, 32–41. 10.1016/j.snb.2019.03.055.

[ref36] Song B. Y.; Zhang M.; Teng Y.; Zhang X. F.; Deng Z. P.; Huo L. H.; Gao S. Selective ppb-level H_2_S sensor for spendable detection of exhaled biomarker and pork freshness at low temperature: Mesoporous SnO_2_ hierarchical architectures derived from waste scallion root. Sens. Actuators B: Chem. 2020, 307, 127662 10.1016/j.snb.2020.127662.

[ref37] Phuoc P. H.; Viet N. N.; Thong L. V.; Hung C. M.; Hoa N. D.; Duy N. V.; Hong H. S.; Hieu N. V. Comparative study on the gas-sensing performance of ZnO/SnO_2_ external and ZnO–SnO_2_ internal heterojunctions for ppb H_2_S and NO_2_ gases detection. Sens. Actuators, B 2021, 334, 129606 10.1016/j.snb.2021.129606.

[ref38] Paolucci V.; De Santis J.; Ricci V.; Lozzi L.; Giorgi G.; Cantalini C. Bidimensional Engineered Amorphous a-SnO_2_ Interfaces: Synthesis and Gas Sensing Response to H_2_S and Humidity. ACS sens. 2022, 7, 2058–2068. 10.1021/acssensors.2c00887. 35757893 PMC9315963

[ref39] Chen T.; Sun J.; Xue N.; Zhang X.; Wang H.; Jiang K.; Zhou T.; Quan H. Co, N-doped GQDs/SnO_2_ mesoporous microspheres exhibit synergistically enhanced gas sensing properties for H_2_S gas detection. J. Mater. Chem. A 2022, 10, 10759–10767. 10.1039/D2TA00837H.

[ref40] Chen T.; Sun J.; Xue N.; Wang W.; Luo Z.; Liang Q.; Zhou T.; Quan H.; Cai H.; Tang K.; Jiang K. Cu-doped SnO_2_/rGO nanocomposites for ultrasensitive H_2_S detection at low temperature. Microsyst. Nanoeng. 2023, 9, 69 10.1038/s41378-023-00517-z. 37260769 PMC10227056

[ref41] Chen Z.; Sivaparthipan C. B.; Muthu B. IoT based smart and intelligent smart city energy optimization. Sustain. Energy Technol. Assess. 2022, 49, 101724 10.1016/j.seta.2021.101724.

[ref42] El-Shamy A. G. New nano-composite based on carbon dots (CDots) decorated magnesium oxide (MgO) nano-particles (CDots@MgO) sensor for high H_2_S gas sensitivity performance. Sens. Actuators B: Chem. 2021, 329, 129154 10.1016/j.snb.2020.129154.

[ref43] Ali F. I.; Mahmoud S. T.; Awwad F.; Greish Y. E.; Abu-Hani A. F. Low power consumption and fast response H_2_S gas sensor based on a chitosan-CuO hybrid nanocomposite thin film. Carbohydr. Polym. 2020, 236, 116064 10.1016/j.carbpol.2020.116064. 32172879

[ref44] Chen K. J.; Lu C. J. A vapor sensor array using multiple localized surface plasmon resonance bands in a single UV–vis spectrum. Talanta 2010, 81, 1670–1675. 10.1016/j.talanta.2010.03.023. 20441956

[ref45] Chiu Y. C.; Deb M.; Liu P. T.; Zan H. W.; Shih Y. R.; Kuo Y.; Ruan D. B.; Gan K. J.; Hsu C. C. Sputtered Ultrathin WO_3_ for Realizing Room-Temperature High-Sensitive NO_2_ Gas Sensors. ACS Appl. Electron. Mater. 2023, 5, 5831–5840. 10.1021/acsaelm.3c00725.

[ref46] Peng S.; Hong P.; Li Y.; Xing X.; Yang Y.; Wang Z.; Zou T.; Wang Y. Pt decorated SnO_2_ nanoparticles for high response CO gas sensor under the low operating temperature. J. Mater. Sci. Mater. Electron. 2019, 30, 3921–3932. 10.1007/s10854-019-00677-7.

[ref47] Geng G.; Chen P.; Guan B.; Liu Y.; Yang C.; Wang N.; Liu M. Sheetlike gold nanostructures/graphene oxide composites via a one-pot green fabrication protocol and their interesting two-stage catalytic behaviors. RSC Adv. 2017, 7, 51838–51846. 10.1039/C7RA11188F.

[ref48] Salehi A.; Ghodsi F. E.; Mazloom J.; Ebrahimi-Koodehi S. Tuning of optical bandgap, conductivity parameters, and PL emissions of SnO_2_: Ni thin films under Ar, N_2_, and O_2_ annealing. Appl. Phys. A: Mater. Sci. Process. 2018, 124, 1–6. 10.1007/s00339-018-2087-2.

[ref49] Deb M.; Chen M. Y.; Chang P. Y.; Li P. H.; Chan M. J.; Tian Y. C.; Yeh P. H.; Soppera O.; Zan H. W. SnO_2_-Based Ultra-Flexible Humidity/Respiratory Sensor for Analysis of Human Breath. Biosensors 2023, 13, 81 10.3390/bios13010081. 36671916 PMC9856198

[ref50] Chen Y.; Fang W.; Liu F.; Kuang K.; Xiao X.; Wei H.; Li M.; He Y. First-principles study of the rectifying properties of Au/SnO_2_ interface. Appl. Surf. Sci. 2023, 637, 157939 10.1016/j.apsusc.2023.157939.

[ref51] Ma X.; Wu X.; Wang Y.; Dai Y. Schottky barrier and band edge engineering via the interfacial structure and strain for the Pt/TiO_2_ heterostructure. Phys. Chem. Chem. Phys. 2017, 19, 18750–18756. 10.1039/C7CP03453A. 28696440

[ref52] Yang X.; Fu H.; Zhang L.; An X.; Xiong S.; Jiang X.; Yu A. Enhanced gas sensing performance based on the fabrication of polycrystalline Ag@TiO_2_ core-shell nanowires. Sens. Actuators B: Chem. 2019, 286, 483–492. 10.1016/j.snb.2019.01.096.

[ref53] Tang W.; Chen Z.; Song Z.; Wang C.; Wan Z. A.; Chan C. L. J.; Chen Z.; Ye W.; Fan Z. Microheater integrated nanotube array gas sensor for parts-per-trillion level gas detection and single sensor-based gas discrimination. ACS Nano 2022, 16, 10968–10978. 10.1021/acsnano.2c03372. 35797450

[ref54] Biring S.; Kolaru R. B. Achieving high response of poly (3-hexylthiophene-2, 5-diyl) molecules to gaseous ammonia using anodic aluminum oxide nanoporous substrate operated under 1 V. Sens. Actuators, B 2022, 373, 132712 10.1016/j.snb.2022.132712.

[ref55] Lee Y. H.; Shin S. I.; Hong J. Y. Investigation of volatile sulfur compound level and halitosis in patients with gingivitis and periodontitis. Sci. Rep. 2023, 13, 13175 10.1038/s41598-023-40391-3. 37580412 PMC10425441

[ref56] Biring S.; Sadhu A. S.; Deb M. An effective optical dual gas sensor for simultaneous detection of oxygen and ammonia. Sensors 2019, 19, 5124 10.3390/s19235124. 31771092 PMC6928993

[ref57] Liu C. Y.; Deb M.; Sadhu A. S.; Karmakar R.; Huang P. T.; Lin Y. N.; Chu C. S.; Pal B. N.; Chang S. H.; Biring S. Resolving cross-sensitivity effect in fluorescence quenching for simultaneously sensing oxygen and ammonia concentrations by an optical dual gas sensor. Sensors 2021, 21, 6940 10.3390/s21206940. 34696153 PMC8539023

[ref58] Zhao C.; Guo X.; Ding Y.; Liang C.; Du B.; Niu W.; Qu W.; Shi Y.; Cong S.; Meng G.; He Y. 2D/0D SnSe_2_/TiO_2_ nanocomposites for trace NO_2_ detection under H_2_S interference at room temperature. Sens. Actuators B: Chem. 2023, 393, 134291 10.1016/j.snb.2023.134291.

[ref59] Hu X.; Zhu Z.; Chen C.; Wen T.; Zhao X.; Xie L. Highly sensitive H_2_S gas sensors based on Pd-doped CuO nanoflowers with low operating temperature. Sens. Actuators B: Chem. 2017, 253, 809–817. 10.1016/j.snb.2017.06.183.

[ref60] He M.; Xie L.; Zhao X.; Hu X.; Li S.; Zhu Z.-G. Highly sensitive and selective H_2_S gas sensors based on flower-like WO_3_/CuO composites operating at low/room temperature. J. Alloys Compd. 2019, 788, 36–43. 10.1016/j.jallcom.2019.01.349.

[ref61] Tian J.; Pan F.; Xue R.; Zhang W.; Fang X.; Liu Q.; Wang Y.; Zhang Z.; Zhang D. A highly sensitive room temperature H_2_S gas sensor based on SnO_2_ multi-tube arrays bio-templated from insect bristles. Dalton Trans. 2015, 44, 7911–7916. 10.1039/C5DT00354G. 25823527

[ref62] Fang G.; Liu Z.; Liu C.; Yao K. L. Room temperature H_2_S sensing properties and mechanism of CeO_2_–SnO_2_ sol–gel thin films. Sens. Actuators B: Chem. 2000, 66, 46–48. 10.1016/S0925-4005(99)00467-0.

[ref63] Kumar A.; Shringi A. K.; Kumar M. RF sputtered CuO anchored SnO_2_ for H_2_S gas sensor. Sens. Actuators, B 2022, 370, 132417 10.1016/j.snb.2022.132417.

[ref64] Mao L.-W.; Zhu L.-Y.; Tao Wu T.; Xu L.; Jin X.-H.; Lu H.-L. Excellent long-term stable H_2_S gas sensor based on Nb_2_O_5_/SnO_2_ core-shell heterostructure nanorods. Appl. Sur. Sci. 2022, 602, 154339 10.1016/j.apsusc.2022.154339.

[ref65] Xiao X.; Liu L.; Ma J.; Ren Y.; Cheng X.; Zhu Y.; Zhao D.; Elzatahry A. A.; Alghamdi A.; Deng Y. Ordered mesoporous tin oxide semiconductors with large pores and crystallized walls for high-performance gas sensing. ACS Appl. Mater. Interfaces 2018, 10, 1871–1880. 10.1021/acsami.7b18830. 29260553

