# Supplementary material for: Achieving Room-Temperature ppb-Level H2S Detection in a Au-SnO2 Sensor with Low Voltage Enhancement Effect
Source: ACS Sens. 2024 May 16;9(9):4568–77. doi: 10.1021/acssensors.4c00105 (PMC11443527; doi:10.1021/acssensors.4c00105)
Supplement: Supplementary file 1 — se4c00105_si_001.pdf [file se4c00105_si_001.pdf]

# Achieving room-temperature ppb-level $H_2S$ detection in Au-$SnO_2$ sensor with low voltage enhancement effect

Moumita Deb [a,b], Chia-Jung Lu [c*], Hsiao-Wen Zan [a,b,*]

[a] Department of Photonics, College of Electrical and Computer Engineering, National Yang Ming Chiao Tung University, 1001, Ta Hsueh Rd., 300 Hsinchu, Taiwan

[b] Department of Photonics, College of Electrical and Computer Engineering, National Chiao Tung University, 1001, Ta Hsueh Rd., 300 Hsinchu, Taiwan.

[c] Department of Chemistry, National Taiwan Normal University,162, Heping East Rd., Section 1, 106 Taipei, Taiwan.

*E-mail: hsiaowen@nycu.edu.tw ;cjlu@ntnu.edu.tw*

## S1. Preparation of Gold (Au) Nanoparticles.

Gold nanoparticles were prepared through the reduction of hydrogen tetrachlororaurate ($HAuCl_4$, Alfa Aesar) in an aqueous phase [44]. A 100 mL aqueous solution of 1 mM $HAuCl_4$ was boiled with vigorous stirring in a round-bottom flask, to which 10 mL of 40 mM citric sodium (Aldrich) was added. The solution was continuously boiled for 10 min, and the color of the solution slowly turned from yellow to purple-red. The solution was cooled at room temperature and stored in a refrigerator at 4 °C for further use. Considering the diameter of AuNPs 18 nm, the concentration of AuNPs in the solution is 5.05 nM.

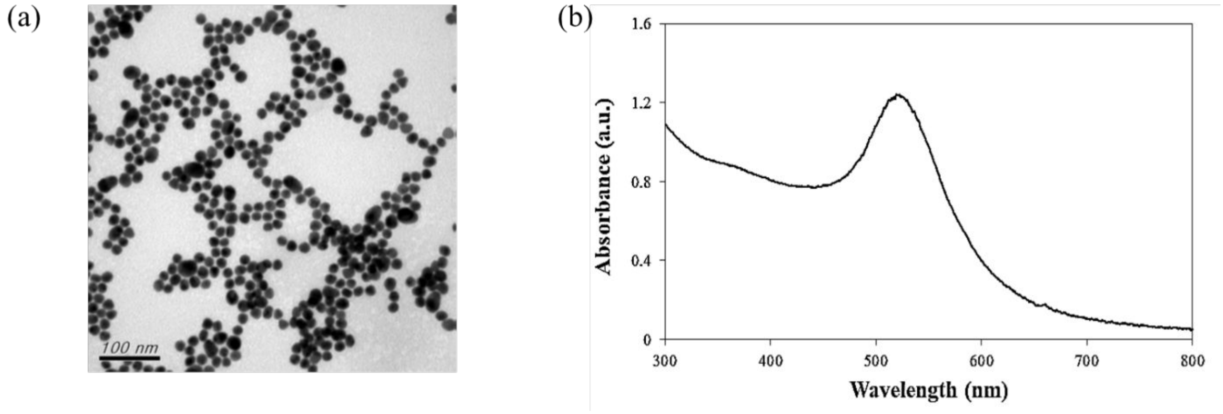


Figure S1. (a) SEM images of Au NPs (b) Absorption spectra of Au NPs

**S2. Instruments:**

ET200 (SANPANY INSTRUMENTS CO., LTD., Taipei, Taiwan) was used to measure thickness (40-60 nm) of $SnO_2$ film after annealing on a glass substrate. UV-Ozone treatment machine (Orient Service Co. Ltd.; TW-UN-URS-500-03) was used to make the surface lyophilic. Thermal evaporator (Thermal resistance evaporation system: Junshang / Glovebox: Mbraun) was used to deposit metal electrode. Spin coater (KYOWARIKEN K-359SD-1) was used to prepare sensing film on glass substrates. Muffle furnace was used to anneal thin film. Atomic force microscopy (AFM, Bruker) was used to analyze surface morphology of film. Scanning electron microscope (FESEM, SU8010) was used to check the structure of sensing film. X-ray diffraction analysis technique (XRD, Bede D1 system) was used to check film crystallinity. X-ray photoelectron spectroscopy (XPS, PHI Quantera II, ULVAC) technique was used to understand the chemical composition of sensing film. Casca software was used to fit XPS energy curve.

**S3. Thin film preparation and sensor fabrication:** Pristine $SnO_2$ was prepared using a simple sol-gel process (Figure 1a). A mixture of ethanol (Honeywell, France) and DI water (1:1) was added to 0.2 M $SnCl_4 \cdot 5H_2O$ (Sigma Aldrich, USA) and stirred magnetically for 24 hours at RT (24±1 °C) and a RH of 60-75% RH. After 24 hours, a 10 wt% AuNPs (10-20 nm)[44] solution was added to the $SnO_2$ solution and subjected to ultra-sonication for 30 minutes. Glasses (Deckglaser, Australia) were cleaned with acetone, alcohol, DI water for 10 min, and dried on a hotplate at 120 °C for 10 min. Therefore, the glass substrates were treated with UV-ozone for 20 minutes. Subsequently, 60 µL of the Au/$SnO_2$ solution was spin-coated onto the glass substrates at 800 rpm for 30 seconds. After coating, the substrates were kept at 60 °C for 10 minutes on a hotplate to evaporate the solution as a pre-annealing treatment. Finally, the film-coated substrates were annealed in a furnace at a rate of 10 °C/min up to 450 °C and held for 2 hours. The furnace was then cooled down to RT, and thermal evaporation was used to deposit a 200 nm Al electrode with a channel width of 1.2 cm and a distance of 200 µm between the two electrodes (Figure 1b). AuNPs information, including SEM images, absorption spectra (Figure S1), and synthesis process (S1), is in the supporting information. The SEM image proves that the size of the AuNPs is between 10 and 20 nm, while the absorption peak at 520 nm confirms the presence of AuNPs. For recording the sensor's current change (at a fixed voltage of 0.5 V) and obtaining the sensing response, an electric signal readout device (Keysight U2722A) was utilized, as explained in the following section.

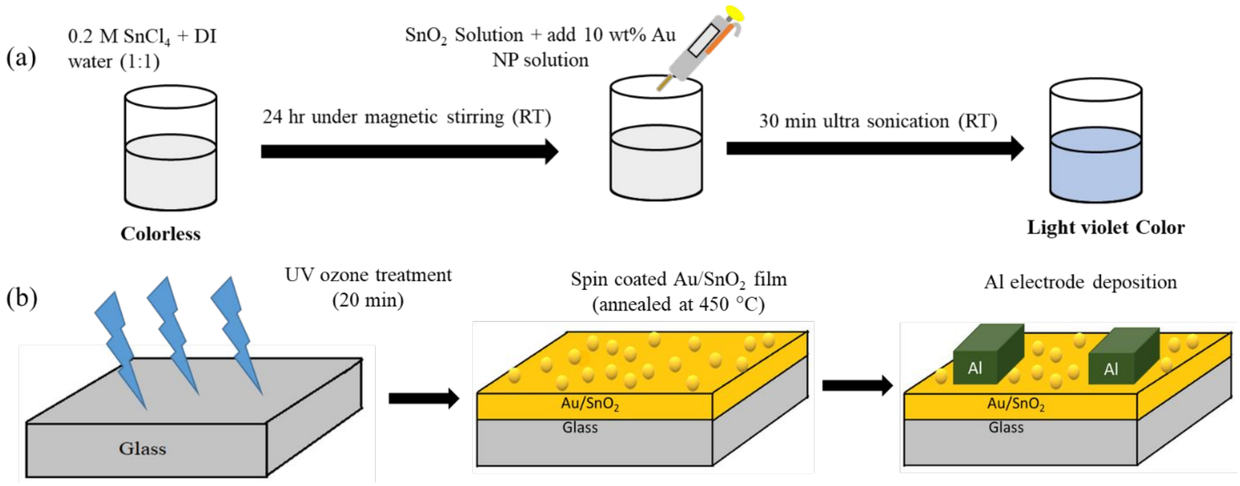


Figure S2. (a) Preparation of Au/$SnO_2$ Solution by sol-gel method (b) Schematic of Au/$SnO_2$-based $H_2S$ sensor.

**S4. Gas sensing measurement system.** For standard gas sensing measurements (Figure S2), we employed various instruments. Initially, an air cylinder connected to a mass flow meter regulated the relative humidity (RH) in the sensing chamber, maintaining it at 17.5±2.5%. The current-voltage (I-V) characteristics of the fabricated devices were assessed using a source meter unit (Tektronix Keithley, 2400) at RT (24±1 °C) and 17.5±2.5% RH. To introduce the target gas ($H_2S$), we utilized a single-channel syringe pump (LSP01-1A). This pump carried $H_2S$ gas, with a 10 ppm concentration, within a syringe, mixing it with background air at adjustable RH and a flow rate of 500 mL/min. The total flow rate at the outlet was monitored using a flow meter to ensure it remained at 500 mL/min. The air mixture with adjustable RH was then introduced into a glass chamber, where the sensor interacted with the injected $H_2S$ gas and the background air mixture at a flow rate of 500 mL/min. By controlling the syringe pump's volume and flow rate, the $H_2S$ concentration in the mixed gas spanned from 2 to 500 ppb. We maintained a constant pressure inside the chamber using flow meter (Dwyer, 1000 cc/min) and a micro pump at the outlet of the sensing chamber. To verify the RH level in the sensing chamber, a thermo hygrometer (CHY 321) was employed.

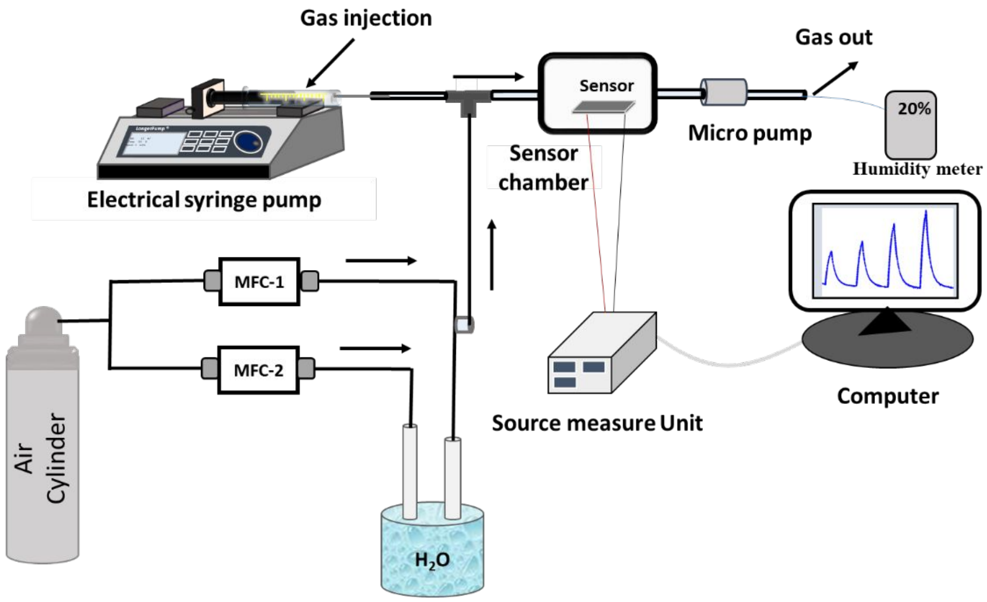


Figure S3. Standard gas sensing measurement system controlling wet/dry air

**S5: Calculation of the dilution of gases:**

All gases were diluted by controlling the flow rate of the entire system. The flow rate and volume of the system were calculated using the following equations:

$$\text{Flow rate of injected gas} = \frac{\text{Injected gas concentration} \times 500\ \text{mL/min}}{\text{Cylinder gas concentration}}$$

For a 30-second injection, the volume of injected gas is given by:

$$\text{Volume of injected gas} = \frac{\text{Flow rate of injected gas}}{2}$$

As example, an analyte of 1 ppm $H_2S$ gas was injected using a syringe pump system, controlling the volume to 25 mL over 30 seconds and maintaining a flow rate of 50 mL/min. Background ambient air was also added to dilute the gas, controlling the overall flow rate at 500 mL/min. Similarly, all other gases were diluted with air by controlling the flow rate. In our syringe pump system, we can control volumes as small as 50 μL (0.05 mL). Tables S3 and S4 provide comprehensive information on the dilution of all analytical gases.

To measure the $N_2$ response, we injected 25 mL of 99% pure $N_2$ gas over 30 seconds, controlling the flow rate at 50 mL/min. Additionally, we introduced background air (20.5% $O_2$ in $N_2$, purity 99.999%) and controlled the overall flow rate to 500 mL/min.

**S6. XRD data.** The crystallinity of both the $SnO_2$ (black color) and Au/$SnO_2$ (green color) films was analyzed using XRD patterns (Figure S4a). The diffraction peaks observed at 26.5º, 38.0º, 44.2º, 64.5º, and 77.6º in the samples are similar to the peaks associated with the (110), (200), (210), (112), and (321) planes of cassiterite $SnO_2$, as indicated by the JCPDS card no. 41-1445 [45]. Additionally, the diffraction peaks of AuNPs are detected at 38.2º, 44.4º, 64.6º, and 77.6º, corresponding well to the (111), (200), (220), (311), and (222) facets of the face-centered cubic (fcc) phase of Au, as stated in the JCPDS no. 04-0784 [46]. Notably, there is no significant shift observed in the diffraction peaks of $SnO_2$ and Au/$SnO_2$ due to the identical peak positions with cassiterite $SnO_2$ and fcc phase of Au. Consequently, this analysis suggests that the Au/$SnO_2$ composite film possesses a good crystalline structure. The energy gap ($E_g$) of $SnO_2$ (4 eV) and Au/$SnO_2$ (3.98 eV) is also shown in the $(\alpha h\nu)^2$ vs Energy curve (Figure S4b), obtained from the absorption spectra using Tauc's equation [47]. Here, α, h, and ν represent the absorption coefficient, Planck's constant, and the frequency of the photons, respectively. This result further confirms the presence of crystalline cassiterite $SnO_2$ with an energy gap of 3.6-4 eV.

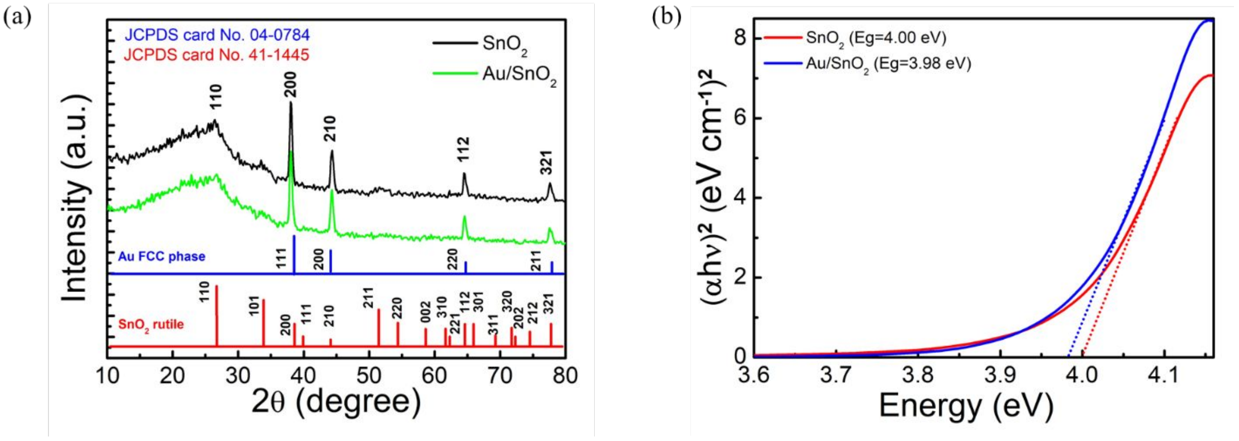


Figure S4. (a) X-ray diffraction pattern (b) band gap analysis from absorption spectra of $SnO_2$ and Au/$SnO_2$ film

**S7. XPS data.** XPS analysis (Figure S5) was employed to confirm the chemical composition and state of $SnO_2$ and Au/$SnO_2$, with all XPS data corrected using the C 1s peak at 284.8 eV [29] as a reference. The non-annealed Au/$SnO_2$ sample initially displayed a Cl-1s peak, indicating the presence of precursor $SnCl_4$ (Figure S5a). However, post-annealing at 450 ºC for 2 hours, the Cl peak vanished in the Au/$SnO_2$ film (Figure S5b), signifying an improved structure following annealing. Notably, significant peaks corresponding to Sn 3d, O 1s, and C were evident in both annealed $SnO_2$ and Au/$SnO_2$ (Figure S5b). The Au 4f peak in Au/$SnO_2$ appeared relatively weak, possibly due to the small amount of added Au (0.1 g Au solution in 1 g of $SnCl_4$), which might hinder the precise analysis of XPS peaks or result in overlap with Sn 2p and Au 4f peaks. In both pure $SnO_2$ and Au/$SnO_2$, the binding energy of the $3d_{5/2}$ and $3d_{3/2}$ peaks was observed at 486.5 and 496.9 eV, and 486.3 and 496.7 eV, respectively (Figure S5c), indicating a spin-orbit splitting of 8.4 eV between $3d_{5/2}$ and $3d_{3/2}$.[29] This splitting suggested the oxidized state of $Sn^{4+}$ in both $SnO_2$ and Au/$SnO_2$. A negative energy shift (around 0.2 eV) was observed in the Sn3d peaks of the Au/$SnO_2$ sample, potentially attributed to Au doping [32]. The peaks at 83.5 eV and 87.1 eV (Figure S5d) confirmed the presence of Au in Au/$SnO_2$. In MOx-based gas sensors, oxygen species' components play a significant role. O 1s XPS spectra typically exhibit three components: lattice oxygen ($O_{lat}$), adsorbed oxygen ($O_{vac}$), and hydroxyl groups ($O_{OH}$). $O_{vac}$ and $O_{OH}$ in sensing materials facilitate oxygen gas induction and create more active adsorption sites, thereby enhancing gas sensing performance. In contrast, $O_{lat}$ remains stable during redox reactions with analyte gases. To comprehend the behavior of O 1s in $SnO_2$ and Au/$SnO_2$, we compared their O 1s XPS peaks (Figure 3a, b). Both samples exhibited O 1s spectra with three components: $O_{lat}$, $O_{vac}$, and $O_{OH}$, which were fitted at approximately 530.2±1 eV, 531.2±1 eV, and 532.6 eV, respectively [32]. In pure $SnO_2$, the relative percentages of $O_{lat}$, $O_{vac}$, and $O_{OH}$ components were approximately 64.92%, 26.49%, and 8.59%, respectively. Upon the addition of AuNPs, the ratio of these components in Au/$SnO_2$ shifted to 58.05%, 37.49%, and 4.46%, respectively.

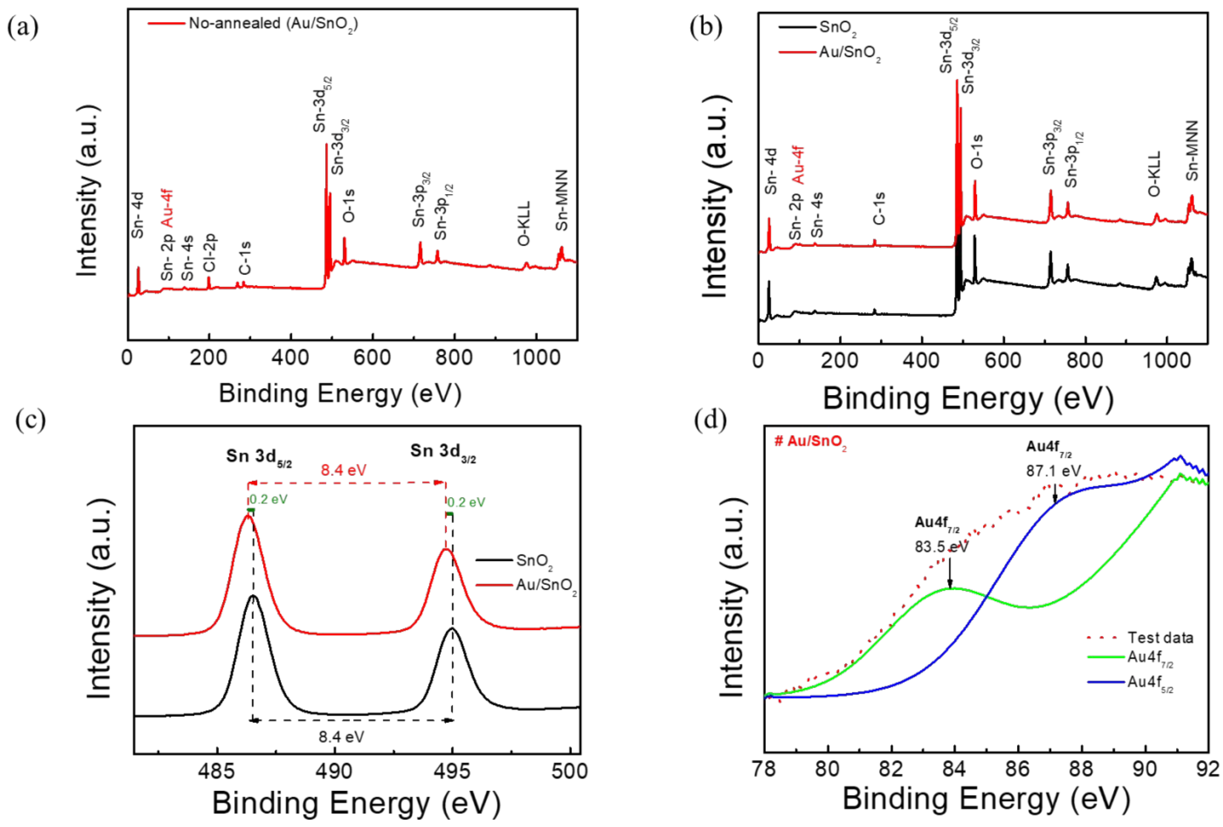


Figure S5. (a) Full XPS spectrum of no-annealed Au/$SnO_2$, and (b) $SnO_2$ and Au/$SnO_2$ film at 450 ºC/2h annealing temperature (c) Sn-3d element spectrum of $SnO_2$ and Au/$SnO_2$ (d) Au-4f element spectra of Au/$SnO_2$.

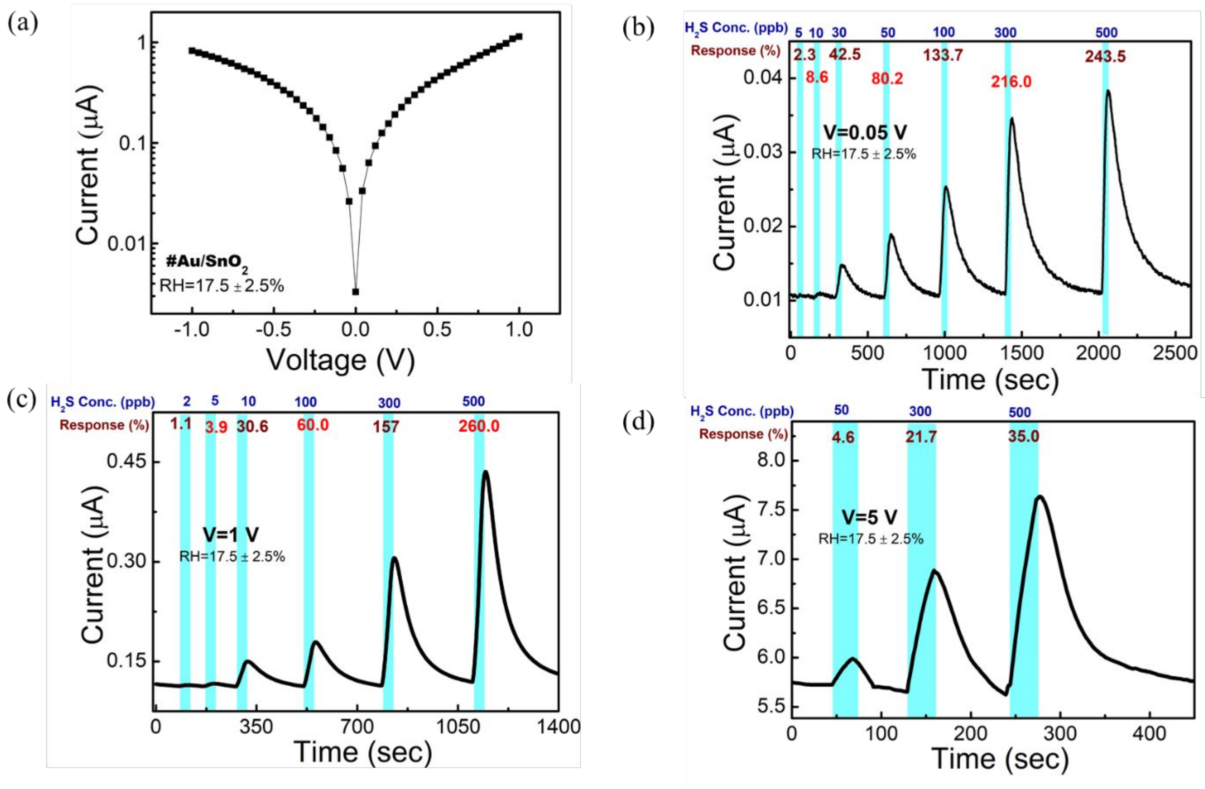


Figure S6. (a) I-V curve of $Au/SnO_2$ based sensor (b) Dynamic response of $H_2S$ gas at operating voltage 0.05 V, (c) 1 V, and (d) 5 V.

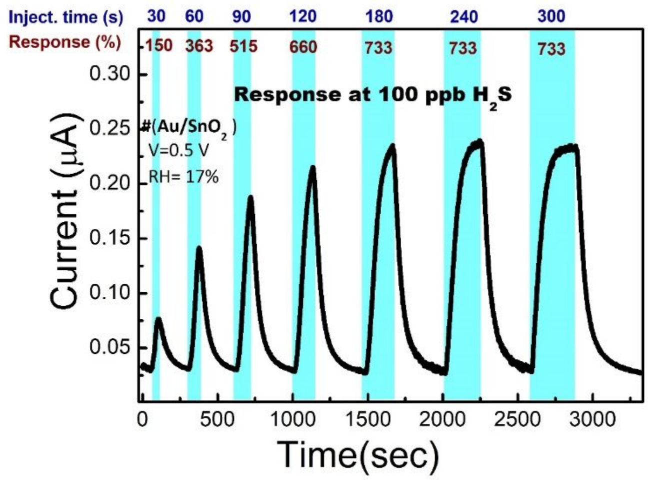


Figure S7. Dynamic real-time gas sensing data recorded under 100 ppb H2S gas concentration with injection times ranging from 30 to 300 seconds.

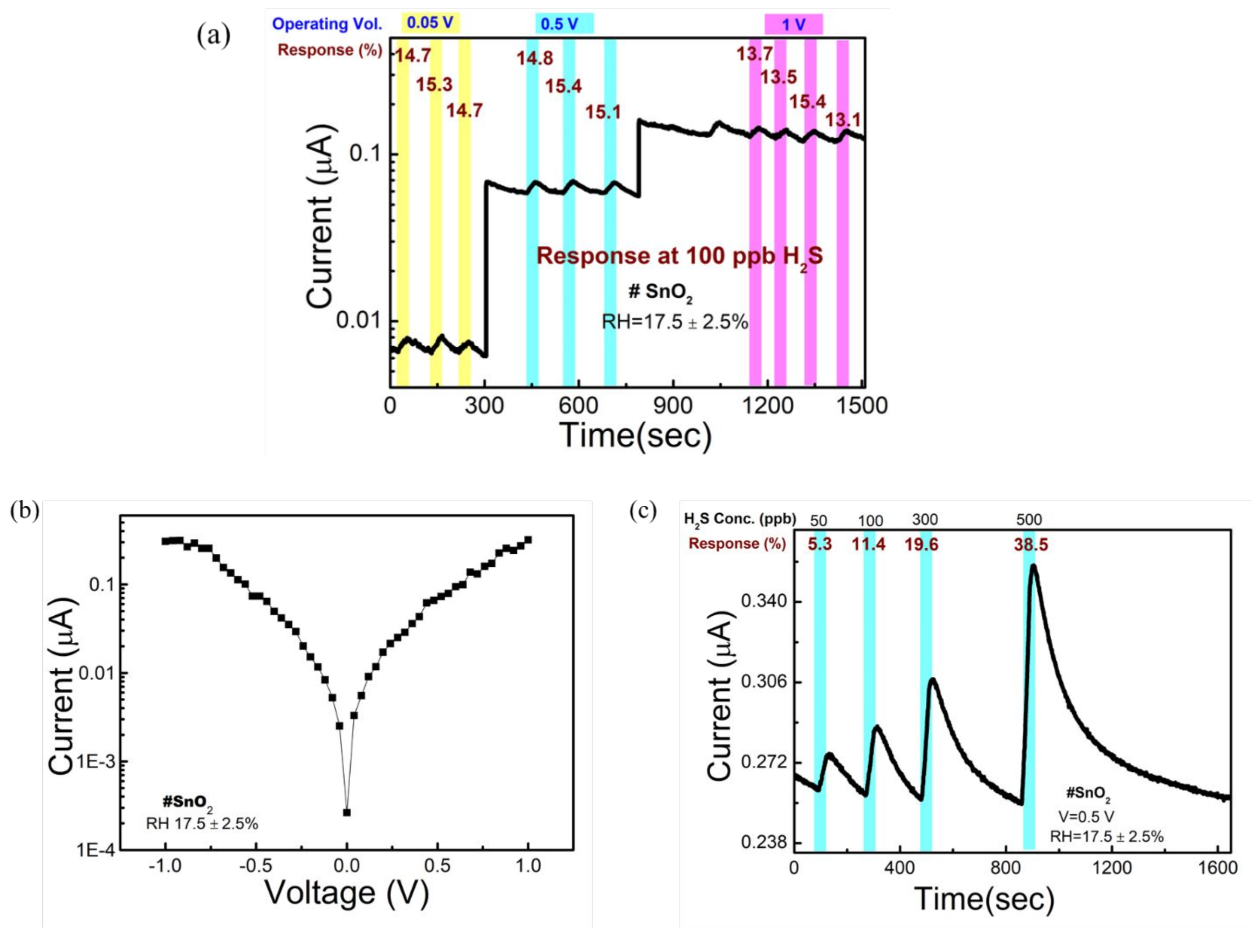

Figure S8. $SnO_2$ based sensor (a) Dynamic response at 100 ppb $H_2S$ gas under different operating voltage. (b) I-V curve, and (c) Dynamic real-time $H_2S$ gas response fin the range of 50 to 500 ppb gas concentration at 0.5 V.

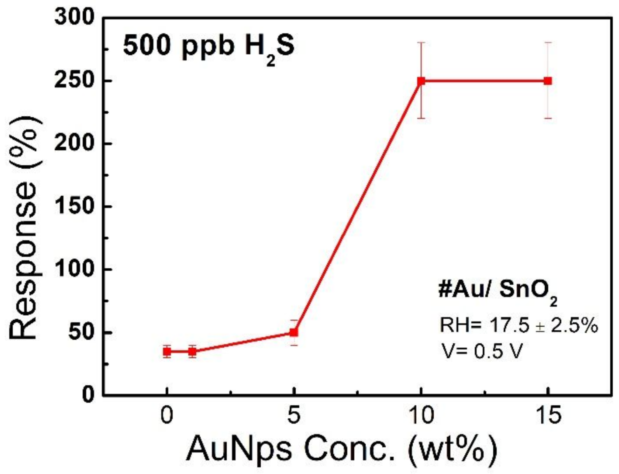


Figure S9. 0.5 V-operated Au/$SnO_2$-based $H_2S$ gas sensor at varying AuNPs concentrations tested with 500 ppb $H_2S$ gas.

**S8. Relative Humidity effect.** The I-V curve (Figure S10a) of the Au/$SnO_2$-based sensor shows increased current with rising RH due to water molecule adsorption on the Au/$SnO_2$ surface. This process creates extra charge carriers in the semiconductor due to the interaction of water molecules with the surface defects and oxygen vacancies of Au/$SnO_2$.[30] The presence of additional charge carrier leads to increase in electrical conductivity and boosting sensor current. The calibration curve (Figure S10b) of $H_2S$ gas response against RH levels indicates that the sensor's response decreases as RH increases. This means that the sensor's ability to detect and respond to $H_2S$ gas is more effective at lower RH levels (between 20% and 30%) compared to higher RH condition (at 50%). The RH-suppression effect was also reported in several prior works.[38,39] The sensor's response to $H_2S$ gas is more pronounced at lower RH levels because the dry surface promotes better interaction between $H_2S$ and the sensor. In contrast, at higher RH levels, the presence of moisture on the sensor's surface creates a barrier that reduces its ability to detect $H_2S$ effectively. This behavior underscores the importance of considering RH conditions when using $H_2S$ sensors, as they can significantly impact sensor performance and response. Real-time dynamic responses to $H_2S$ gas at various RH levels (20%, 30%, and 50%) at 0.5 V (Figure S10c-e) depicted that the current level increases with rising RH, whereas the response decreases with higher RH. It is worth noting that conventional $SnO_2$-based $H_2S$ gas sensors often require high operating energy inputs (Table S1, Table S2), such as UV light irradiation or elevated operating temperatures, to mitigate the impact of water molecule adsorption or humidity on the sensor's surface under high humid conditions. In contrast, our room-temperature and low-humidity-operated $H_2S$ sensor demonstrates that operating under reduced humidity conditions yields outstanding sensing performance that can rival traditional high-temperature-operated processes.

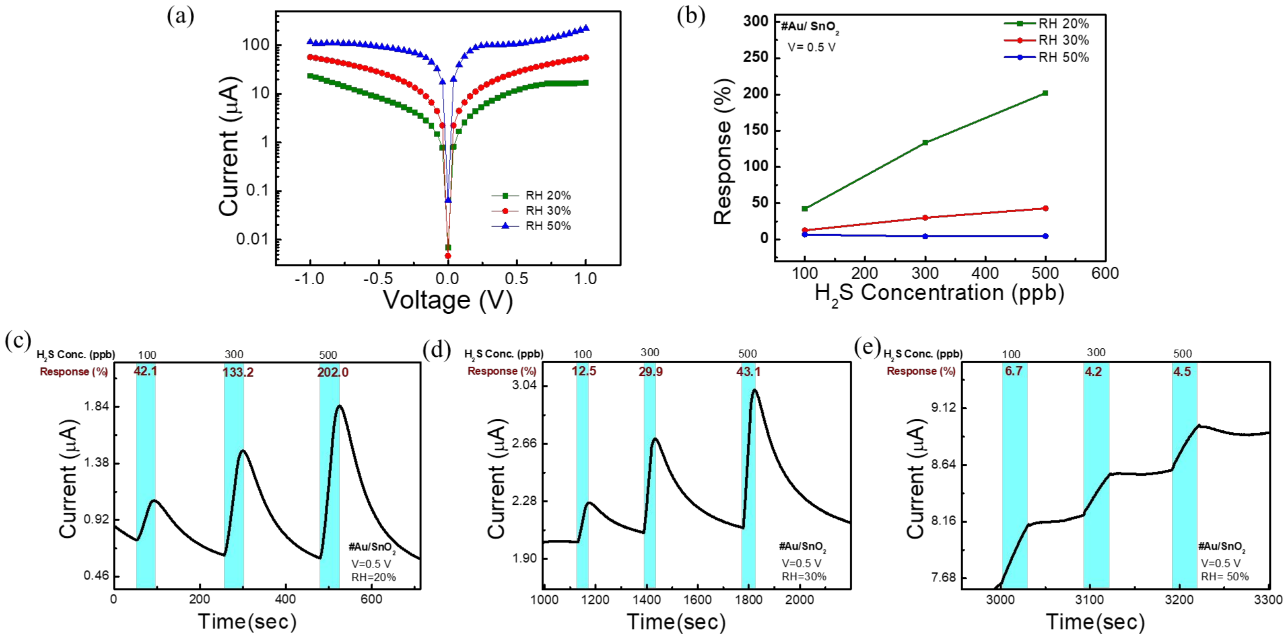


Figure S10. (a) I-V curve, (b) Response vs $H_2S$ gas concentration calibration curve. The dynamic response of Au/$SnO_2$ based $H_2S$ gas sensor under different RH (c) 20% (d) 30% (e) 50%.

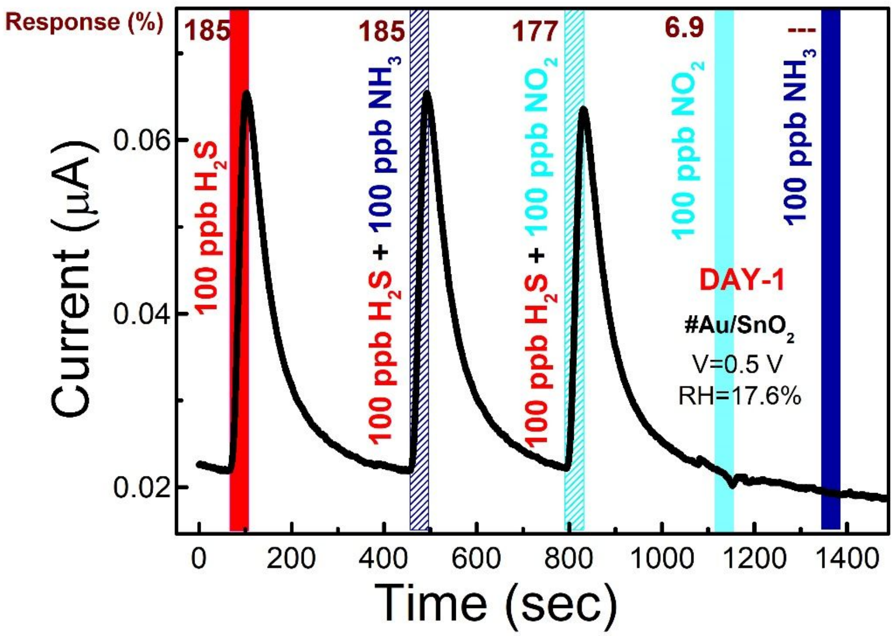


Figure S11. The sensor under mixed gas condition, such as reducing/reducing gas (100 ppb $H_2S$+ 100 ppb $NH_3$) and reducing/oxidizing gas (100 ppb $H_2S$ + 100 ppb $NO_2$).

**S9 Life-time.** The sensor demonstrates a good response of 265.3% at room temperature on day 1 at 500 ppb. Conversely, on day 2, no response was observed. This could be attributed to two factors: prolonged exposure to $H_2S$ resulting in the accumulation of $SO_2$ gas, hindering $H_2S$ attachment to the Au/$SnO_2$ surface, and the presence of water molecules obstructing $H_2S$ access. Consequently, a decline in $H_2S$ gas response occurred on the second day. Subsequent thermal heating (50°C/15 min) restored sensor functionality, yielding a response of 73.2%. This suggests that heating facilitates the desorption of $SO_2$ or water molecules, allowing $H_2S$ to reattach to the sensing surface. Therefore, future improvements in sensor lifetime could be achieved through sample heating. More future work is needed to develop efficient annealing conditions without degrading the sensor response.

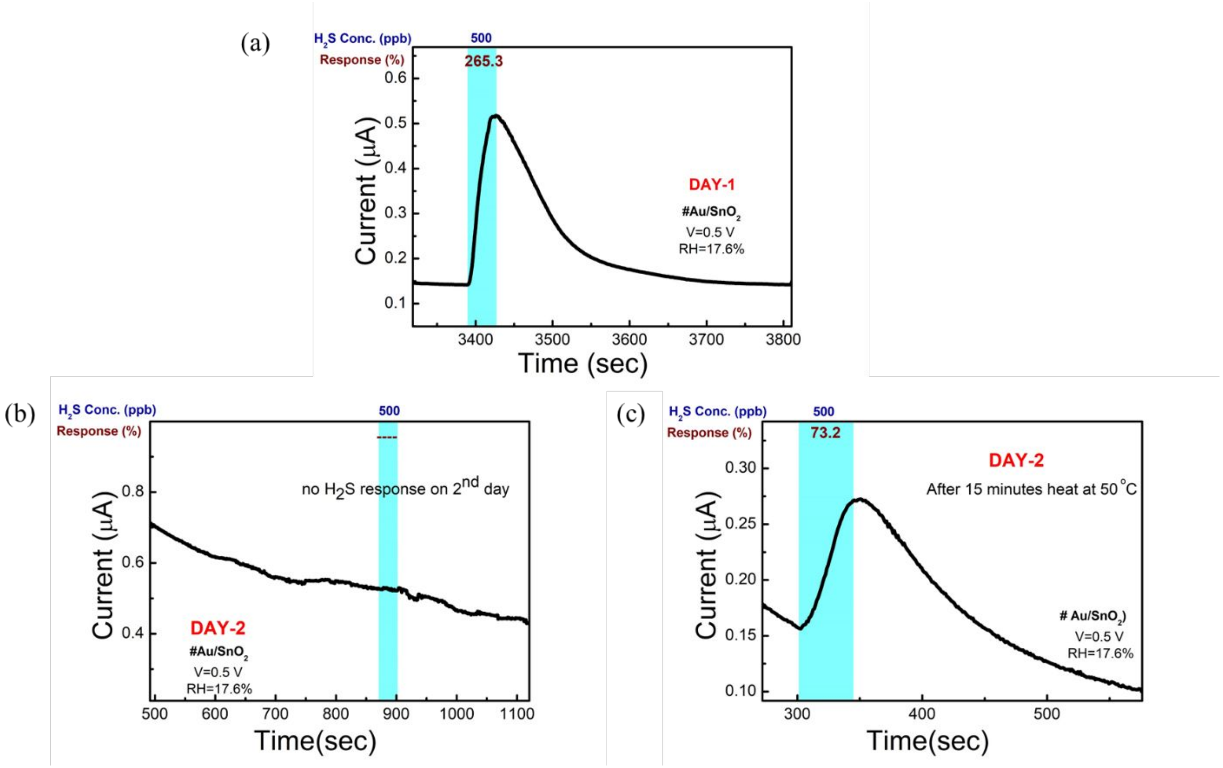


Figure S12. $H_2S$ response at 500 ppb gas concentration on (b) 1st day, (b) 2nd day and, (c) after heating on hotplate (50 ºC/ 15 min) on 2nd day.

Table S1: Au/$SnO_2$ based gas sensors

| **Material** | **Working tempe-rature (°C)** | **RH (%)** | **Analyte gas** | **Gas conc. (ppm)** | **Response** | **Response/ recovery time** | **LOD (ppb) experimentally** | **Ref.** |
|---|---|---|---|---|---|---|---|---|
| Macroporous Au/$SnO_2$ | RT (UV-365) | 0 - 49 | $NO_2$ | 0.2 | 145 | 125/100 sec | 7 | [29] |
| Au/$SnO_2$ | 25-300 | --- | CO | 100 | 67.4% | 3/77 sec | -- | [30] |
| Au/$SnO_2$ hollow microspheres | 240 | 50±5 | Ethanol | 200 | 50 | 4/48 sec | 2500 (6.65 calculated) | [31] |
| MOFs-derived Au/$SnO_2$ | 240 | 32 | Acetone | 100 | 18.2 | 5/66s | 1000 (40 calculated) | [32] |
| Au loaded ZnO/$SnO_2$ | 350 | 60 - 90 | $H_2S$ | 1 | 73.3 | 36/786 sec | 100 | [33] |
| Au/$SnO_2$ nanotubes | 300 | 90 | $H_2S$ | 5 | 34 | ---- | 30 | [34] |
| **Flower-petal-like Au/$SnO_2$ nanostructure** | **RT** | **17.5±2.5** | **$H_2S$** | **0.5** | **~270%** | **30/126 sec** | **2 (0.28 calculated)** | **This work** |

Table S2: Recent $SnO_2$ composite based $H_2S$ gas sensor with ppb level LOD

| Material | Working temperature (°C) | RH (%) | Gas conc. (ppm) | Response | Response/ recovery time | LOD (ppb) practically | Ref. |
|---|---|---|---|---|---|---|---|
| **Au/$SnO_2$ nanotubes** | 300 | 90 | 5 | 34 | --- | 30 | [34] |
| **$SnO_2$/rGO/PANI nanocomposite** | RT | --- | 5 | 76.2% | 80/88 sec | 50 | [35] |
| **Mesoporous $SnO_2$** | 92 | 11-94 | 0.1<br>100 | 2.0<br>106.9 | 56/230 sec<br>5/3100 sec | 0.5 | [36] |
| **ZnO-$SnO_2$ nanofibers** | 350 | --- | 1 | 317 | ~10/450 sec | 10 (0.04 calculated) | [37] |
| **Au loaded ZnO/$SnO_2$ Nanofibers** | 350 | 60 - 90 | 1 | 73.3 | 36/786 sec | 100 | [33] |
| **Amorphous a-$SnO_2$ 2D flakes** | 100 | 10-80 | 1000 | 2.4 | 9/32 min | 210 | [38] |
| **Co, N-GQDs/$SnO_2$ Mesoporous microsphere** | 260 | 33 | 100 | 37.3 | 3/13 sec | 50 | [39] |
| **Cu-$SnO_2$/rGO nanocomposites** | 120 | 55% | 2<br>10 | 180<br>1415.7 | 31/253 sec<br>120/15 sec | 50 | [40] |
| **Flower-petal-like Au/$SnO_2$ nanostructure** | **RT** | **17.5±2.5** | **0.5** | **~270%** | **30/126 sec** | **2 (0.28 calculated)** | **This work** |

Table S3: The applied flow rate and volume for 1 ppm different analyte gases

| Analyte gas | Cylinder gas concentration (ppm) | Concentration of injected analyte gas (ppm) | Flow rate of analytic gas (mL/min) | Volume of injected gas in 30 sec (mL) |
|---|---|---|---|---|
| **$H_2S$** | 10 | 1 | 50 | 25 |
| **NO** | 100 | 1 | 5 | 2.5 |
| **$NO_2$** | 200 | 1 | 2.5 | 1.25 |
| **Acetone** | 100 | 1 | 5 | 2.5 |
| **$NH_3$** | 100 | 1 | 5 | 2.5 |
| **CO** | 25 | 1 | 20 | 10 |

Table S4: The applied flow rate and volume for $H_2S$ gas in different gas concentration.

| Concentration of injected analyte gas (ppb) | Flow rate of analytic gas (mL/min) | Volume of injected gas in 30 sec (mL) |
|---|---|---|
| **500** | 25 | 12.5 |
| **300** | 15 | 7.5 |
| **100** | 5 | 2.5 |
| **10** | 0.5 | 0.25 |
| **2** | 0.1 | 0.05 |

Table S5: Response/recovery time alongside gas response at different $H_2S$ concentrations under different operating voltage (3 set data).

| **Operating voltage (V)** | **Concentration (ppb)** | **Response time ($T_{90}$) (sec)** | **Recovery time ($T_{90}$) (sec)** | **Max. response (%)** |
|---|---|---|---|---|
| **0.05** | 5 | 32 | 40±5 | 3±1 |
| | 50 | 32 | 170±10 | 80±5 |
| | 100 | 32 | 220±20 | 125±10 |
| | 300 | 32 | 240±40 | 196±20 |
| | 500 | 32 | 280±50 | 220±30 |
| **0.5** | 5 | 32 | 70±10 | 18±1 |
| | 50 | 32 | 130±20 | 125±5 |
| | 100 | 32 | 150±20 | 160±20 |
| | 300 | 32 | 160±40 | 210±20 |
| | 500 | 32 | 175±50 | 250±30 |
| **1.0** | 5 | 32 | 40±5 | 4±1 |
| | 50 | 32 | 80±10 | 30±3 |
| | 100 | 32 | 120±20 | 60±10 |
| | 300 | 32 | 150±40 | 155±20 |
| | 500 | 32 | 160±50 | 220±30 |
| **5.0** | 50 | 30 | 20±2 | 5±1 |
| | 100 | 30 | 25±5 | 10±2 |
| | 300 | 30 | 60±10 | 22±3 |
| | 500 | 30 | 120±30 | 40±5 |

Table S6: Maximum response and response/recovery time of Au/$SnO_2$-based sensor under 100 ppb $H_2S$ gas at different

| **Injection time (sec)** | **Max. Response (%)** | **Response time ($\tau_{90}$) (sec)** | **Recovery time ($\tau_{90}$) (sec)** |
|---|---|---|---|
| **30** | 150 | 40 | 130 |
| **60** | 363 | 54 | 120 |
| **90** | 515 | 69 | 130 |
| **120** | 660 | 97 | 130 |
| **180** | 733 | 110 | 130 |
| **240** | 733 | 110 | 130 |
| **300** | 733 | 110 | 130 |
